# MatriGrid^®^ Based Biological Morphologies: Tools for 3D Cell Culturing

**DOI:** 10.3390/bioengineering9050220

**Published:** 2022-05-20

**Authors:** Patrick Mai, Jörg Hampl, Martin Baca, Dana Brauer, Sukhdeep Singh, Frank Weise, Justyna Borowiec, André Schmidt, Johanna Merle Küstner, Maren Klett, Michael Gebinoga, Insa S. Schroeder, Udo R. Markert, Felix Glahn, Berit Schumann, Diana Eckstein, Andreas Schober

**Affiliations:** 1 Department of Nano-Biosystems Engineering, Institute of Chemistry and Biotechnology, Ilmenau University of Technology, 98693 Ilmenau, Germany; maipatrick@outlook.de (P.M.); martin.baca@emse.fr (M.B.); dana.brauer@tu-ilmenau.de (D.B.); sukhdeep.singh@tu-ilmenau.de (S.S.); frank.weise@tu-ilmenau.de (F.W.); justyna.borowiec@tu-ilmenau.de (J.B.); merle-johanna.kuestner@tu-ilmenau.de (J.M.K.); maren.klett@tu-ilmenau.de (M.K.); michael.gebinoga@tu-ilmenau.de (M.G.); 2 Placenta Lab, Department of Obstetrics, Jena University Hospital, 07747 Jena, Germany; andre.schmidt@med.uni-jena.de (A.S.); markert@med.uni-jena.de (U.R.M.); 3 Biophysics Division, GSI Helmholtzzentrum für Schwerionenforschung, 64291 Darmstadt, Germany; i.schroeder@gsi.de; 4 Institute of Environmental Toxicology, Martin-Luther-University Halle-Wittenberg, 06097 Halle, Germany; felix.glahn@uk-halle.de (F.G.); berit.schumann@uk-halle.de (B.S.); diana.eckstein@uk-halle.de (D.E.)

**Keywords:** scaffolds for 3D cell culture, hepatocyte culture, scaffold manufacturing, manipulation of organoids, stem cell niches, neurons and cerebral bodies, automated cell culturing unit, 3D micropattern technique, microcontact printing, thermoforming, cell adhesion, extracellular matrix

## Abstract

Recent trends in 3D cell culturing has placed organotypic tissue models at another level. Now, not only is the microenvironment at the cynosure of this research, but rather, microscopic geometrical parameters are also decisive for mimicking a tissue model. Over the years, technologies such as micromachining, 3D printing, and hydrogels are making the foundation of this field. However, mimicking the topography of a particular tissue-relevant substrate can be achieved relatively simply with so-called template or morphology transfer techniques. Over the last 15 years, in one such research venture, we have been investigating a micro thermoforming technique as a facile tool for generating bioinspired topographies. We call them MatriGrid^®^s. In this research account, we summarize our learning outcome from this technique in terms of the influence of 3D micro morphologies on different cell cultures that we have tested in our laboratory. An integral part of this research is the evolution of unavoidable aspects such as possible label-free sensing and fluidic automatization. The development in the research field is also documented in this account.

## 1. Introduction

A continuously rising number of literature sources claiming the similarities of 3D cell culturing to in vivo data shows the self-depicting importance of mimicking the three-dimensional microenvironment for cell culture [1]. The topic of 3D cell culturing featuring different aspects of their advantages is excellently reviewed [2,3,4]. The design and construction of oligocellular agglomerates or embroid bodies, such as cell aggregates or tissue-like oligocellular structures, are driven by different biological and technological trends in stem cell research [5]. Various techniques, such as 3D bioprinting [6], micromachining [7], hydrogel assembly [8], and microfluidics [9], are being developed day by day to obtain better ECM materials. Microfluidic approaches such as the hanging drop method [10] have led to embroid bodies as a starting point for the evolution of organoids, whereas 3D bioprinting with different types of cells embedded in, e.g., a bio ink as a type of scaffold-based approach is another way to come to organ-like structures [11]. Many of these techniques use gel-based approaches, which often vary in their composition and additionally limit the nutrient and oxygen supply [12]; therefore, more and more gel-free systems, such as anchorage-dependent cell culture [13], fiber-based scaffolds (either electro spun [14] or hollow fibers [15]), hanging drop culture [16,17], dielectrophoresis [18], micropillar [19], 2PP fabricated scaffolds [20,21], and microwell 3D culture [22], are in demand. 

The hanging drop culture is perhaps the most widely used 3D cell culture method because it produces spheroids with uniform morphology; the operation is convenient and low cost. However, the workload is significant, and the high throughput operation is difficult to achieve. Efforts to resolve these disadvantages have resulted in commercial hanging drop plates in 96- and 384-well formats [23,24]. A. Ganguli et al. developed a silicon-based hanging drop microarray suitable for high throughput applications and compatible with high-resolution confocal microscopy [25]. A new hanging drop approach for producing consistent droplet volume was created by combining the hanging drop array with a fluidic system [26]. Consistent droplet volume is achieved by the spontaneously pulling of the cell suspension toward an internal chamber, which is caused by the pressure difference across the hanging drop array. A perfused hanging drop array based on a PMMA microfluidic chip was developed by Shu-Wei Huang et al. [27]. The chip design uses taper tubes for increased droplet stability and an enhanced liquid exchange rate.

In a comparative study, two cancer cell lines, MCF7 and OVCAR8, were used to grow spheroids using three different methods: the hanging drop plate, liquid overlay on an ultra-low attachment (ULA) plate, and liquid overlay on a ULA plate with rotating mixing [28]. The last method produced significantly more chemoresistant spheroids with higher amounts of deposited extracellular matrix.

Additionally, bottomless well plates are used for the hanging drop technique to form a droplet of medium within each well that is big enough for cells to aggregate but small enough to not fall off while cultured [4].

Other techniques to generate such self-assembled cell aggregates (spheroids) include low adhesion plates, v-shaped plates, and magnetic cell levitation [4,29]. The advantage of low adhesion plates or v-shaped plates compared to hanging drop plates is the avoidance of transfer of spheroids after their formation to a different plate for further culturing or experiments as these plates have a larger volume. Cell aggregation is prevented by using polystyrene plates that are treated with hydrophilic or hydrophobic coatings such as the non-adherent polymer poly-HEMA [30] or natural polymers such as agarose [31]. A new complex technique for generating spheroids is magnetic cell levitation. In this technique, cells are loaded with magnetic nanoparticles and levitated towards the air–liquid interface using a magnetic field. Magnetic cell levitation has been successfully used to produce monocellular and multicellular spheroids [32,33,34,35,36]. 

Compared to the spheroid building technique, culturing cells in 3D microcontainers brings the additional advantage of restricting the culture to growth within particular geometrical and biochemical boundaries. These, in turn, affect the cellular behavior of organoid cell culture. There is no doubt that all these features and parameters play an important role in the understanding (and the redesign) of biological systems. Higher macroscopic organisms consist of biological components on the microscale and nanoscale, so the starting point in this analysis is to consider all the geometric scales involved in the system. We have introduced the term “biotechnical multiscale engineering” for a methodological approach to designing artificial hybrids of biological and technological systems [37]. Hereby, many biological systems are analyzed, ranging from the sub-nanometer elements such as cell adhesion molecules to micron-sized capillaries and the macroscopic dimensions and functionality of whole organs. Thus, for better control over culturing space, shape, diffusion parameters, and imaging, transparent thermoplastics such as polycarbonate (PC) [38,39], poly methyl methacrylate (PMMA) [40,41,42], polystyrene (PS) [43], and cyclic olefin polymers or copolymers (COP/COC) [44,45] are being explored. The general resistance of thermoplastics toward changes in temperature, pressure, and high chemical stability makes them ideal for mass production [46].

Based on the idea that each specific organ-like cell agglomerate also needs a specific geometrical shape, we have developed tools and techniques for the construction of a family of polycarbonate substrate scaffolds that we call MatriGrid^®^s. These scaffolds are suitable not only for cell culturing but also for the manipulation and evolution of embroid bodies, for mimicking stem cell niches, or for control of the behavior of tissue slices. Some of these scaffold-based approaches use polymeric scaffolds for shaping the evolving oligocellular agglomerates. Scaffold-based approaches allow us not only to define the shape of the agglomerates but also to control biophysical and mechanical properties such as stiffness, shear stress, and nutritious flow if integrated into lab-on-a-chip devices or bioreactors [47].

The technique is based on template or morphology transfer technology, where the forming of thermoplastic (bio)-polymers allows us to design some complex geometries. This opens promising perspectives for the mimicking and bio-fabrication of free-form complex morphologies for the construction of more native and tissue-like microarchitectures [37] or manipulation tools [48]. This toolbox also includes methods of micrometer-scale biochemical or topographical patterning, which are commonly used to guide cell attachment and growth. By use of our advanced 3D microcontact printing (3DμCP) [49], predesigned microstructures can be fabricated. This includes fluidic channels with different depths and widths that contain biochemical cues or even more complex patterns.

Recently, we described a good example of the biotechnical multiscaling approach using template transfer technology by biomimetic reconstruction of the hematopoietic stem cell niche for in vitro amplification of human hematopoietic stem cells [50,51,52]. The main geometric features of a microtome intersection from the bone marrow (BM) of a human long bone were retrieved by image processing. The extracted features were adapted to construct photolithographic masks. This way, tools and molds for polymeric reproduction were designed (following the structure replication in different polymeric embodiments on an MTP footprint) and, finally, used for cell amplification experiments [50,51].

Apart from the scaffold design, organized fluidics is the most demanding part of such complex organotypic 3D models. Unlike a conventional cell culture, a special readout mechanism is required for the qualification of the culture parameters without manual intervention in mostly static and passive fluidics [53,54,55]. A big improvement is the dynamic (perfused) fluidic systems, with possibilities to enhance nutrition and metabolite transport in 3D cell culture [56,57,58]. It is also known that the flow direction has a significant influence on cell proliferation, which ultimately guides vascularization [59,60,61]. Furthermore, in a recent study of 3D MatriGrid^®^ environments (static and perfused) for the culturing of neuroblastoma cancer cells, this was analyzed in detail and compared with neuroblastoma tissues and 2D culture, showing that anti-cancer drug treatments in 3D are closer to the real-world situation than a 2D culture [62]. In perfused culture systems for long-term experiments, it is desirable that the necessary media exchange and the sampling of probes for analytics are carried out in an automated way.

What is needed for analytical approaches for 3D in vitro systems? First of all, devices/scaffolds that bring the cells into a 3D shape and behavior. Second, the devices in which such scaffolds with the cell agglomerates are preferably handled. Third, an open interface to all traditional laboratory equipment, with the possibility of easily transferring the scaffolds to readers, microscopes, analytical devices, etc. It would also be desirable to integrate analytical functionality directly into the 3D cell culture devices, but this would result in complicated lab-on-chip experimental setups. Although they might be very fruitful, such solutions would still be expensive and not standardized in biological medicinal laboratories. In a parallel paper in this issue, we described a case study for such an integrated approach of a flow-through ELISA, together with our automatic bioreactor unit, based on commercially available components.

In the following sections, we describe our tools, techniques, and designs, with selected technical and biological examples, that use different polymeric scaffolds with adapted geometry and patterning to meet the 3D cell culture requirements for individual organs.

In the first part, we give an overview of the MatriGrid^®^ scaffold family and describe the scaffolding techniques based on thermoforming tools, together with methods for functionalizing such substrates. In the following chapter, we introduce tools for fluidics and analytics, such as the microbioreactor device, the 3D-MEA device (for measuring electroactive neuronal cell agglomerates), and the automatic sampling drug application unit. Then, we report different species of MatriGrid^®^s with their morphology, function, and biological results. The following section describes the long-term measurements of the hepatic cell culture and the biological results achieved. We close the description of our account of research with a classification with respect to contextualization and a discussion of further perspectives of technological development. 

## 2. Overview

Advanced cell culturing technologies should not only fulfill the demand of oligocellular cell populations, such as in real organs, but they should also meet physical, engineering, and economic aspects to make them suitable for a wide range of applications. However, many advanced cell culturing approaches developed in the past have maintained their individual and different characteristics [63]. Therefore, for scaffold-based 3D culturing devices, we consider the following engineering constraints and conditions desirable:

- All the scaffolds and scaffold holders should be compatible with the MTP footprint to support laboratory automation standards.
- Scaffold production should allow cost-efficient mass production.
- Scaffold production should be scalable with respect to morphology and design (e.g., pore size).
- If required, it should be possible to introduce and modify pores and channels specifically in order to achieve high diffusion gradients, flow-through, or active material transport.
- For the devices adopting MTP and other footprints, compatibility with common laboratory equipment should be preferred as much as possible. This means that wherever possible, an open interface to readers, microscopes, etc., should be chosen. This includes aspects such as easy transfer, easy handling of covers, transparency of material, etc.
- The platform design should be modular, and the building blocks should be able to connect to each other; for example:
  ○ Possibility of stacking scaffolds in an MTP well or in a microbioreactor.
  ○ Possibility of cascading single-unit micro bioreactors with optional fluid addition, e.g., to make drug administration or dilution of metabolic products possible.

Nevertheless, we were seeking a general method that could offer a feasible and rapid technique with the potential of integrating most of the information, data, and parameters in the field of advanced cell culturing systems. It is surely difficult to meet all biological, geometrical, and physiological constraints within a single approach. To make it more feasible from the (bio)-physical point of view, one should confine the limits of a system to particular characteristics.

We have chosen geometrical aspects of an organ as a major boundary condition because it can offer cellular confinement relevant to its native tissue. For example, in order to mimic the geometrical aspects of a liver-like advanced cell culture system, a cell culture medium flow rate of approximately 20 µL/min is required to adequately supply a liver lobule (approx. 2 × 10^5^ cells) with oxygen. This value is derived from the oxygen demand of the cells. For a 3D cell cluster, the maximum size that can be supplied by diffusion is approx. 300 µm (see Supplementary Information: O_2_ consumption of a liver lobule).

**Figure 1:**
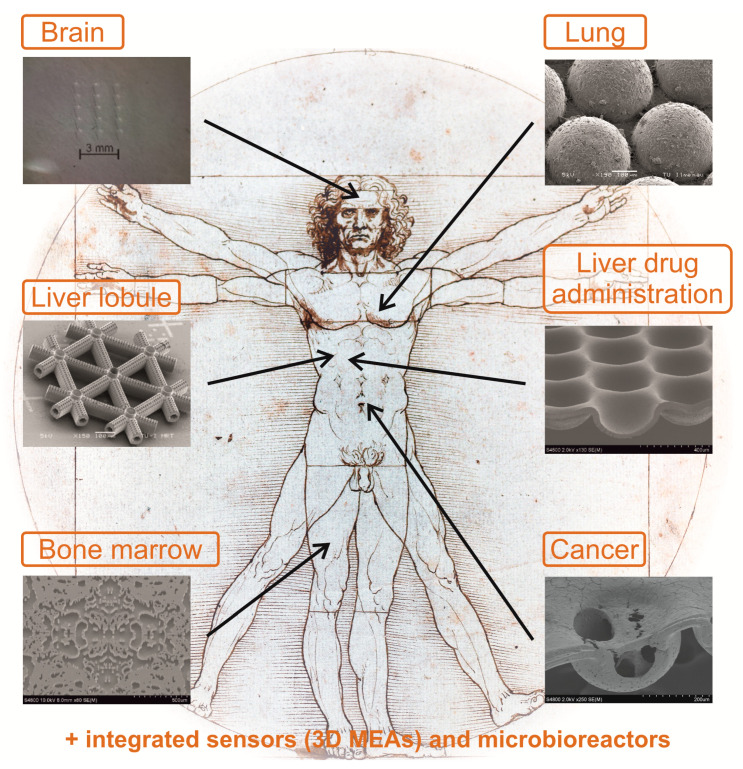
An overview of different applications of MatriGrid^®^s for mimicking different organs. In this account of research, we select the brain, the liver, and the lung as applications of the MatriGrid^®^ scaffold for culturing specific cells from these organs. The liver lobules [20], bone marrow [50], and application to cancer research [62] are given elsewhere.

The same line of engineering arguments could be held for every native tissue model. From our point of view, aiming mainly for the geometrical aspect of different organs is mimicked easily by 3D microstructured morphologies, which have high chances for cost-effective production by template transfer technologies and, in turn, can facilitate the widespread utilization of native-tissue-inspired morphologies in science and industry. Based on our research philosophy, various geometrical approaches constrained to individual organs are shown in Figure 1.

As depicted in Figure 1, in principle, it is possible to mimic the geometrical aspects of almost every tissue. However, it might be misleading to generally compare advanced cell culturing systems using different approaches, materials, and techniques. There might be advantages of one system that can overcome another. If we focus, for example, on liver cell culture, for which there are, to date, well-explored advanced cell culturing systems, one can witness the relative and complimentary advantages of each system with respect to one another (see Table 1). In the following section, our approach to mimicking various tissue morphologies is explained.

### The MatriGrid^®^-Family—Overview

Three-dimensional (3D) cell cultures are becoming increasingly important as this method of cell culturing better mimics tissue physiology in multicellular organisms [84]. The 3D cell cultures can be classified according to scaffold-free and scaffold-based systems [84]. In scaffold-free 3D cell cultures, single cells are seeded in suspension culture. By preventing the cells from adhering to the walls of the cell culture vessel, the cells combine to form multicellular aggregates. These cell aggregates, formed by self-assembly, are commonly referred to as spheroids [85]. Spheroids have a variety of properties, such as ideal physiological cell–cell interactions, the formation of their own extracellular matrix (ECM) components, and better cell–ECM interactions [86]. On the other hand, organoids can be distinguished if 3D cell cultures from embryonic or primary stem cells, as well as primary tissue, show organ functions after self-assembly [87].

On the other hand, in scaffold-based 3D cell cultures, the cells are embedded in a matrix, and the properties of the cells growing there are determined by the chemical and physical properties of the scaffold itself. The scaffolds are designed to promote cell adhesion and cell–cell and cell–matrix interactions. Furthermore, adequate transport of nutrients and gases should be enabled to support cell growth and avoid toxicity [88]. In addition, 3D structures facilitate tissue-specific differentiation [89]. Technically, scaffold-based 3D cell cultures can be based on soft hydrogel or solid polymer structures. A variety of natural (fibrin, collagen, hyaluronic acid, silk, and gelatin) and synthetic (synthetic polymers, titanium, bioactive glasses, and peptides) materials are used as soft scaffolds, which are manufactured using nearly equal large numbers of manufacturing processes. On the other hand, polymer-based solid scaffolds have proven to be particularly suitable due to their properties, such as high porosity, small pore sizes, biodegradability, and mechanical properties [84]. Solid-scaffold-based 3D cell culturing systems provide mechanical support to the cells growing in a 3D environment, due to which the cellular outcome of such cultures is closer to the in vivo situation. Our research group has focused on polymer-based 3D scaffolds, tuned through the mentioned micro thermoforming technique that is described in detail in the following chapter. The tools and materials that the micro thermoforming technique depends on provide nearly unlimited possibilities to construct geometries of various designs, ranging from simple micro containers to complex bio-mimicking topographies. 

The first ideas of scaffold-based containers for cell culturing were guided by the philosophy of forcing the cells to grow in a 3D manner or forming intercellular 3D contacts defined by the geometric shape in such a way that the cells inside the cell agglomerates would not become necrotic [90,91,92]. This was achieved by selecting proper scaffold geometry with dimensions that allowed nutritious supply to the cells by diffusion or perfusion through porous scaffold structures. In both cases, this may be implemented either by active fluidics or passive means, such as the diffusion or convection of the media. One has to consider not only the biophysical constraints but also the given technical limits of the physical properties of the material, micromachining, and tooling. In principle, with micro thermoforming technology, microstructures with dimensions down to 10 µm are feasible. However, it depends upon the specific structure and thickness of the source material. For example, in the case of MatriGrid^®^, 50 µm thick foil was stretched into a micro mold up to 300 µm depth. Beyond these dimensions, the cavities will rupture due to the lack of material strength. Therefore, the first MatriGrid^®^ design was constructed as a scaffold with defined porous and non-porous regions, which allowed a controlled flow of the media through a 3D culture of pure hepatic cells [37,93,94,95]. Further development and applications are also possible by applying oligocellular cell mixtures in such a cavity; for instance, applications to cancer research have become possible [62].

Another idea is to mimic the real fluidic structure of an organ and then guide the different cell species to the right place on the scaffold for adhesion. The structures of MatriGrid^®^s are modeled according to the functional units of respective organs and their specific geometrical characteristics (biotechnical multiscale engineering) [20]. Using this idea, the first MatriGrid^®^ design was created for directed oligocellular cultures, which was inspired by the morphology of the lung [96,97]. Following the idea of constructing real geometric environments for cell culturing, we developed a scaffold for mimicking the blood stem niche in bone marrow, which we call BMGrid^®^ [50,51,52]. Similarly, to develop MatriGrid^®^ for growing neurons, we evolved our micro-container-based scaffold into specialized linear structures for the growth and handling of neurons and spheroids. In another study, a line- and space-based 3D scaffold was developed to roughly mimic capillary structures as a base to approach the morphology of a liver lobule [98].

In the following section, we describe the learning outcome from the MatriGrid^®^ scaffold family: MatriGrid^®^ with applications to liver cells and the oligocellular lung alveoli model, NeuroGrid^®^ for application to neuronal cells, and TissGrid^®^ for application to placenta explants.

## 3. Micro Thermoforming and Functionalization of MatriGrid^®^s

### 3.1. Micro Thermoforming

Most polymeric scaffolds are accessible through microscopical assessment, which makes them advantageous to inorganic/organic scaffolds or scaffolds derived from hydrogels. In addition, they are compatible with a wide range of processing technologies that provide access to low-cost production. Polymeric foils are also suitable for thermoforming; therefore, they are an attractive raw material for manufacturing polymeric 3D cell culturing scaffolds. For biological applications in cell culture, the scaffolds need to have accurate and reproducible properties; otherwise, experimental results are hardly comparable or even meaningless. Porous membranes offer advanced functionalities as they allow for the continuous perfusion of the cells through the scaffold’s micro pores, leading to an enhanced supply of nutrients and oxygen. In the following chapters, various selected scaffolds and their applications are presented, which were basically produced by the technology described below.

The micro thermoforming technology presented here produces reproducible scaffolds with precisely defined properties. Due to the single-stage manufacturing process, efficient production of scaffolds is possible through parallelization. To ensure the high homogeneity of the scaffolds, aspects of permanent quality control have been integrated. However, a major disadvantage of the ordinary thermoforming process is the inability to form porous materials due to pressure equalization through the pores during the processing of the material. This disadvantage can be overcome by adding an additional, non-porous support film that transfers the forming pressure to the porous film under isostatic conditions. In this process, a so-called polymer sandwich is formed. This one-step process results in the effective and parallel production of scaffolds with precisely defined and reproducible properties [99]. The carrier film can then be easily removed from the structured porous membrane. Boundary conditions for the production of the sandwich are the result of the forming temperature of the porous membrane, the material composition of the sandwich, and the thickness of the two films to ensure an optimal working process. A thermoforming machine (Wickert presstech, 76829 Landau i.d.Pf., Germany) specially designed for this application provides both vacuum and high pressure by means of integrated pumps and compressors. All these modules are integrated into the compact housing of the machine.

**Figure 2:**
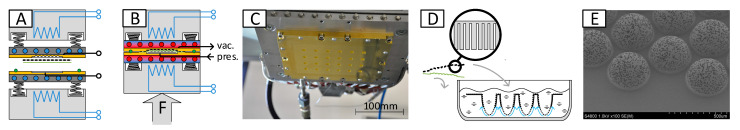
(**A**) Preparation of the semi-porous foil and protection layer made of FEP foil; (**B**) closing of the tool and pressure impulse to stretch foils into the mold, beginning of cool down; (**C**) micro structured mold for 24 scaffolds on MTP footprint; (**D**) wet etching of the microstructures to receive porous cavities only, (**E**) resulting semi-porous scaffold with a spatial distribution of pores only inside the cavities.

Separation of the heating and cooling plates by a two-stage mold design enables fast process sequences. Until the film stack is pressure-loaded, the heating plates are separated from the cooling block by insulated springs and guide rails. The cooling block is made of aluminum, which has a large heat capacity compared to the heating plate. The thin but rigid heating plate is made of special tool steel with a high elongation at break. Before the gas pressure is applied, the press closes the remaining 1 mm gap between the cooling block and the heating plate. Now, a counterforce to the gas pressure can be built up. This counterforce can be adjusted by varying the gas pressure. Guide blocks were mounted to align the heating plate and cooling block and to allow for different thermal expansions of the materials used. This setup provides good mold guidance and short cycle times. Figure 2 shows a scheme with a description of the manufacturing steps, used tools, and fabricated scaffold.

Isostatic molding technology has many advantages, such as uniform force distribution, easy force generation, and independence from the wedge error of the two sides of the tool. Tools can be made not only from a variety of established materials (e.g., metal, ceramics, semiconductors, or plastics), but it is also possible to use soft materials as tool dies [100]. Tools can thus be manufactured by a variety of manufacturing processes. Examples are conventional processes such as milling and EDM, but etching techniques used in microstructuring and rapid prototyping technologies can also be used.

A disadvantage is that the technique is mainly used to form thin materials. The isostatic principle is also disadvantageous in the forming of porous materials due to the use of a fluid as a force transmitter. The applied fluid pressure would immediately penetrate the membrane, depending on the pore geometry and the parameters of the fluid. Thus, the mapping of the mold geometry would no longer be possible.

In principle, there are two basic ways to process yet-porous substrates by thermoforming. One established method, as described above, is to use a transfer membrane. It allows the direct use of the porous film. A novel method was demonstrated by Giselbrecht et al., who processed films prepared before ion irradiation by thermoforming [92]. Later, the formed structures were etched to obtain porous microstructures. The advantage of this method is the ease of process adjustment.

**Figure 3:**
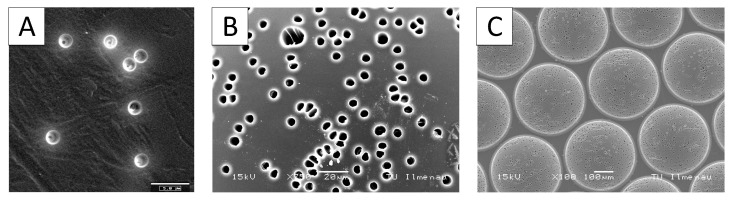
(**A**) Formation of bottlenecks due to annealing in the center of the pores, (**B**) etched pores in PC without prior heat treatment of the foil; (**C**) microstructured scaffold produced to the method described above, with bottleneck-free pores on the structured cavities only.

One problem with this method is the annealing of the pre-irradiated ion tracks due to the thermal effects during the heating and cooling cycle. A detailed description of the annealing process can be found in Skehon et al. [101]. This leads to an uneven pore geometry. Recent research shows the appearance of membrane-like structures in the center of the holes, so-called apertures, as a result of the annealing of the ion tracks. These act like bottlenecks and increase the flow resistance of the entire pore enormously. Figure 3A,B show a comparison of healed and native porous PC film. Healing occurred after processing the film, according to the time scale that is standard for modified embossing machines.

Afterward, the film can be made porous by etching under alkaline conditions. Compared to the native and untreated film, the membrane with previous heat treatment shows a centered constriction in the etched pore. The heat treatment is a part of the thermoforming process in which the film is heated from room temperature to forming temperature (about 160 °C for polycarbonate), formed, and then cooled down to room temperature in a few minutes. This cycle time is caused by the large thermal mass of the heating plates in a conventional embossing machine. Additionally, the installed heating power is small compared to the mass of the heating plate. Due to these process-related limitations, the flow resistance of the foil increases enormously. Unfortunately, such defects are not visible during optical microscopy inspection of the transparent foil and can only be investigated using SEM images and cross-sections. To overcome this disadvantage, design and technological efforts must be taken to shorten the cycle times. Figure 3C shows an etched film with a short heat treatment before etching, according to the method described earlier and seen in Figure 2. Here, no annealing of the ion tracks can be seen due to the short cycle times. It illustrates the need for fully integrated and adapted processing of these special films with tailored thermoforming technology. Depending on the compact and robust machine design, mold design, and geometry, the heat treatment during the process significantly influences the manufacturing results. The polymers provide a good opportunity for additional treatment. Shaped polymers can be coated by ECM material or functionalized by chemical methods.

### 3.2. Microcontact Printing and Chemical Functionalization

Microcontact printing has proved to be a facile technique for patterned cell growth [102]. However, with the present microcontact printing techniques, there is no provision to create microscopic geometrical barriers along with chemical patterning. In other words, technical means of transferring biochemical molecules to the pre-structured micro geometries in a precise, reproducible, and high-throughput manner are limited [103,104,105]. Therefore, the applicability of conventional microcontact printing remains restricted when complex 3D surfaces, e.g., channels or tubular structures with defined chemical and topographical micropatterns, are desirable [103]. 

We have developed a 3-dimensional microcontact printing (3D µCP) technique within the context of thermoforming that is capable of structuring microtopographies on the surface and the precise transfer of the extracellular matrix (ECM) proteins into the obtained geometries simultaneously [49]. By combining the advantages of microcontact printing with microthermoforming to synchronize the chemical and topological patterning in one step, the extension of the scope of 2D surface patterning to the 3D surface is possible. Conventional stamps made from polydimethylsiloxane (PDMS) [106] can be used. In brief, a stamp with a target pattern was selectively inked with biomolecules and subsequently used as a mold for microthermoforming. Therefore, microstructuring and chemical patterning could be performed at the same time. During thermoforming, a polymer film is heated up to the thermoplastic state and formed under gas pressure onto a mold to replicate its topography [107]. Microthermoforming is highly reproducible, efficient for mass production, and works for most thermoplastics polymers, including biodegradable polymers used in tissue engineering as well as porous and permeable polymer films [108]. We chose polycarbonate membranes as a substrate because they are commonly used for cell culture applications, are highly transparent, and are available in different thicknesses and pore sizes [109]. Our process has been validated by microscopic measurements and fluorescence staining. 

**Figure 4:**
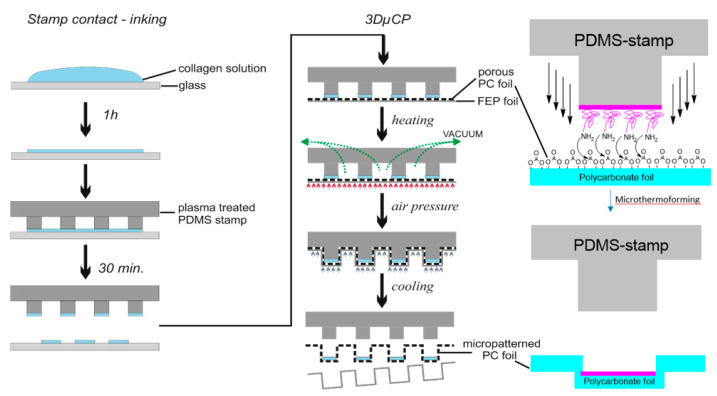
Scheme of channel fabrication using the 3D μCP method and chemical functionalization on the bottom of the channel.

Among the various polymeric materials explored for solid 3D scaffold formation, polycarbonate is prominently suitable for thermoforming applications. Even in the case of 3D microcontact printing, it has a good chemical affinity to biopolymers such as collagen. The most probable reason for the chemical binding of the amine-containing biopolymers with polycarbonate is carbamate formation (Figure 4). Inspired by the tendency of covalent bond formation between amine and carbonate, we explored various terminal diamines and polyamines [110] to chemically functionalize the polycarbonate surface. Even the post thermoforming functionalization of polycarbonate surface was stable under cell culture conditions. Such aminized polycarbonate surfaces were explored to attach biologically interesting molecules and dyes [110]. Even photochromic dyes such as donor-acceptor Stenhouse adducts were explored as visible-light-assisted photoswitches on polycarbonate surfaces [111,112]. 

## 4. Tools for Fluidics and Analytics—The Bioreactors 

### 4.1. µ Inserts

**Figure 5:**
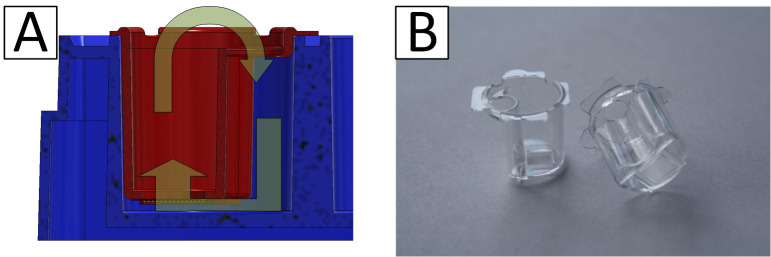
(**A**) Scheme of a semi-active system with the possible flow direction of media shown by arrows. Shown in blue is a standard MTP well; red is the insert with a scaffold attached to the bottom; (**B**) manufactured semi-active system with a MatriGrid^®^ scaffold.

The step from well-known and established 2D cell cultures to advanced 3D cell cultures is related to many innovations in culture management, working methods, and equipment. For many users, these challenges are associated with incalculable risks, which is one of the reasons why the application of 3D cell cultures in research and development has a high barrier to entry [113]. The so-called semi-active systems were developed in order to find a simple way for us to get into this field [114]. They combine the advantages of the well-known 2D cell culture with the essential possibilities offered by a 3D culture. These semi-active systems are based on units that can be used in the familiar 24-well microtiter plates. The laboratory handling of 24-well plates is part of common training and ensures comfortable manipulation by the user. Similar developments have been initiated in recent years by leading manufacturers of laboratory products [115]. In our case, within the inserts, different microstructured scaffolds can be used. This possibility is unique to our system. In this way, two fluidically separated compartments are formed in one well. If the microstructured scaffolds are porous, a certain flow through the scaffold and the cell culture contained in it to the other compartment can be achieved by a slight hydrostatic pressure generated by different levels of media in both compartments. This way, a very simple variant of an active perfusion of the cell culture is possible. Of course, the flow rate is not constant, as it would be with an active pump, but by recurrent pipetting of the liquid between the compartments, a volatile flow through the membrane can be generated. Due to the design of the systems, this recurring pipetting can be easily and quickly done by a robot. A good diffusive supply of the cell culture is also possible using an appropriate membrane. In addition, the use of high gradients of pharmaceutically active medium components and the partial segregation of cell products into the medium are possible. The handling is simple, and the systems can be easily inserted into the well and removed with tweezers. The inserts position themselves automatically, which even allows the use of multipettes or automatic transfer stations and pipetting robots. The lid of an MTP can be used conventionally, which prevents the evaporation of media and cross-contamination and allows convenient handling and transport. Due to the small distance between the scaffolds and the original well bottom, the cell culture can also be observed via inverted microscopy. Maximum compatibility with established laboratory methods is guaranteed. The systems can be manufactured cost-efficiently as disposables by injection molding. Thus, a simple and inexpensive entry into the capabilities of 3D cell culture is possible. As an example, the capability for air–liquid cultures is presented in the article. In addition to the ease of use of the systems, their application allows the characterization and observation of cell cultures in an intermediate-like stage between conventional 2D culture and sophisticated 3D cultures with active perfusion in microbioreactor systems. Thus, they provide a valuable source of data for linking both types of culture and, thereby, lead to a better understanding of the models. Figure 5 shows the schematic structure of a system and a picture of manufactured inserts. 

### 4.2. The Bioreactor Family

**Figure 6:**
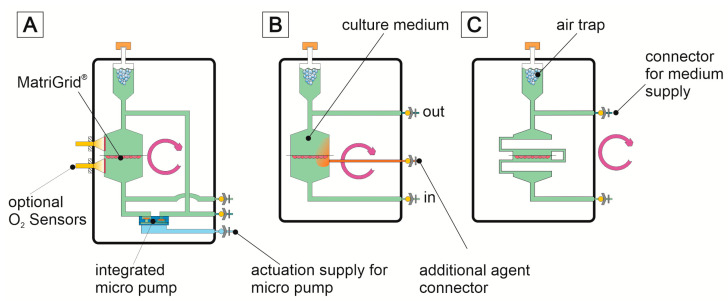
(**A**) Microbioreactor with integrated micropump. In this system, the cell culture medium can be pumped internally via the micropump or externally via a peristaltic pump; (**B**) with this connection variant, it is possible to generate an active substance gradient across the MatriGrid^®^; (**C**) in this type of microbioreactor, the MatriGrid^®^s is supplied with nutrients via superfusion. This greatly reduces the shear forces on the cells in the microcavities.

For the 3D culturing of cells, it is advantageous to replace a passive nutrient supply (diffusion) with an active one. Many different systems and concepts for 3D cell culturing have already been published. A concise overview can be found here [40,116]. A direct comparison of these systems is usually not possible because they are based on very different concepts and aims to be solved. However, there are systems/concepts that are very similar in functionality to ours, e.g., [117]. Our MatriGrid^®^s can be used for passive 3D culture as well as for active perfusion. For active perfusion of the MatriGrid^®^s, we have developed a number of micro bioreactors for various applications during the last 15 years. These systems have an internal volume of about 1.0 to 1.5 mL. The volume is adapted for the well size of a 24-well plate. The optimal flow rate depends on the cell type. There are two important factors that are mutually exclusive: on one hand, the O_2_ transport and, on the other hand, the shear stress on the cells. O_2_ transport is directly proportional to the flow rate. However, many cells do not tolerate large shear forces but have high O_2_ consumption. A compromise must be found here in order to optimally supply the cells but also not to overload them with shear forces (HepG2 25 µL/min, HepaRG 12 µL/min). In most cases, the cell culture medium is pumped in a closed loop. Different microbioreactor variants can be chosen for this purpose. In the simplest case, a peristaltic pump is used for perfusion. We have also integrated commercially available micropumps (Bartels MP6) or micropumps based on our own developments [118] for this purpose. Additionally, syringe pumps can also be utilized, and we used them to simulate time-dependent drug courses (MMT MDSP3f, MMT GmbH, Siegen, Germany). Figure 6 shows the fluidic scheme of the microbioreactors.

**Figure 7:**
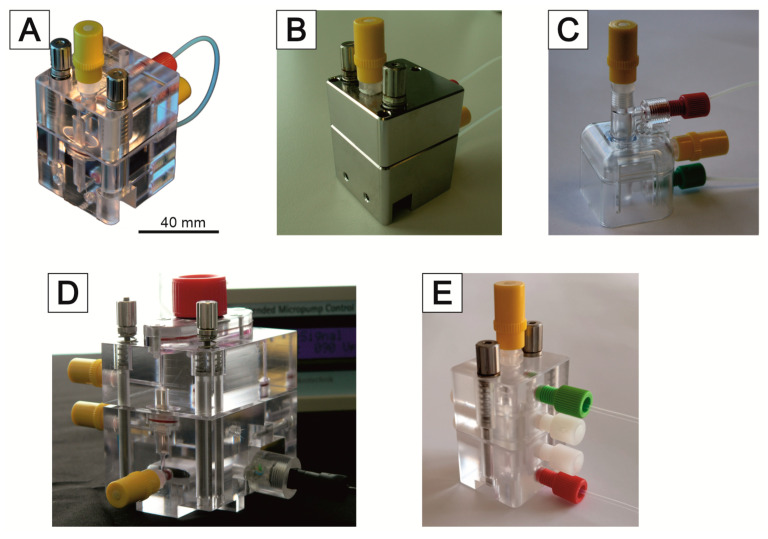
(**A**) Microbioreactor Model 4 in PC version; (**B**) Model 4 in stainless steel; (**C**) Model 5 manufactured using injection molding; (**D**) Model 7 with integrated micro pump; (**E**) Model 6 microbioreactor for perfusion and superfusion.

Like the MatriGrid^®^, the bioreactors are made of transparent polycarbonate (PC). This also allows a view into the system. To minimize absorption effects by the walls of fluidic channels, one type of reactor was also made of inert steel (1.4404). The first versions of the reactors were manufactured by milling. Because this is laborious and expensive, these systems are intended for multiple use. The material is suitable for sterilization by superheated steam and with ethanol. In order to provide a sufficient quantity of microbioreactors for laboratory work, we have developed a variant (Figure 7C) that is manufactured by injection molding and can be used inexpensively as a disposable product. PC was again used as the material because it performed well with the milled microbioreactors. This bioreactor consists of only two parts, which can be closed with an integrated snap closure. All microbioreactors are equipped with 1/4-28 thread connectors to allow easy and flexible connectivity (P-205, Upchurch Scientific Inc., Oak Harbor, Washington, DC, USA). The systems are vented via infusion plugs. Likewise, active ingredients can still be added via these plugs, or samples can be taken during operation (IN-Stopfen, PZN: 02133082, B.Braun, Melsungen, Germany). 

**Figure 8:**
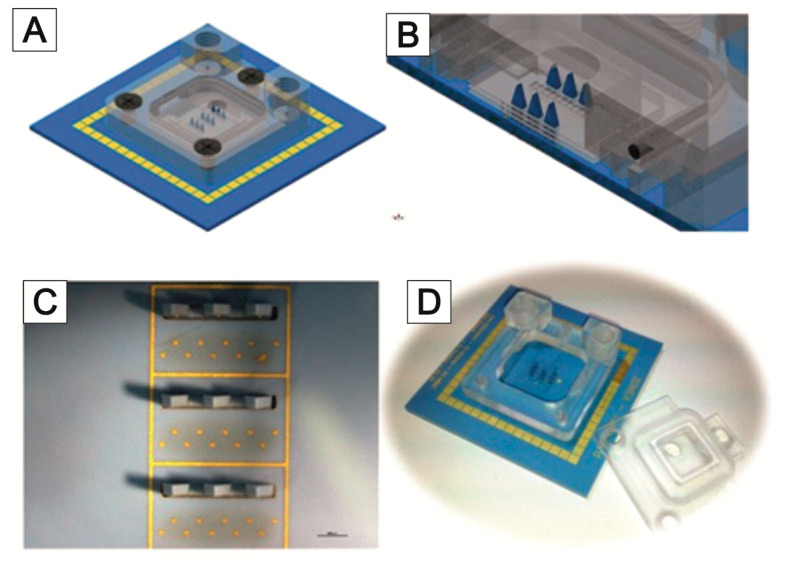
(**A**) 3D MEA with cell culture chamber; (**B**) stacked cell carrier foils on the needle electrodes; (**C**) sensor surface of the 3D MEA; (**D**) real 3D MEA system with a specially adapted polymeric micro bioreactor with dimensions of 30 × 30 mm.

Figure 7 shows a selection of variants manufactured and used. In most cases, we use the system pictured in Figure 8C. If we need information about the dissolved O_2_ concentration, the system of Figure 8A,D can be used. A sensor system can be integrated to measure dissolved oxygen concentration and consumption. This allows the real-time monitoring of the cells [119]. The reactor in Figure 7E has a very modular design. Inserts for perfusion or superfusion can be placed into the upper or lower part. This reactor is mostly suitable for experiments requiring superfusion. Due to this flexible design, it is also possible to realize lower or upper cross flows to the main flow. In this way, it is possible to generate gradients of active substances via the MatriGrid^®^ (Figure 7B).

The analysis of the cells in such systems occurs mostly by the analysis of the supernatants or exchanged media using standard equipment. The other possibility is an on-demand analysis by integrated sensors, as described above, or the integration of sensors in the fluidic circuit, as described by Baca et al. in this issue or in the way of Zhang et al. [120]. More and more controlled parameters have recently been integrated into a self-standing system; this leads to complex and sophisticated systems, as shown by de Bournonville et al. [121]. From our point of view, it is more beneficial to introduce optical and biochemical read-out parameters, thus keeping the system compatible with standard laboratory read-out systems.

However, electroactive cells should be analyzed not only by optical-based assays but also by direct measurement of the electroactivity of such cells, respectively, in 3D cultures. Therefore, such devices have to be developed, which is the subject of the next section.

### 4.3. Analytical Unit/the 3D MEA

While extracellular recordings by microelectrode arrays (MEAs) are subject to the attenuation and temporal filtering of electrical signals, a relatively large number of excitable cells can be stimulated and recorded simultaneously. Due to the marker-free and non-invasive recording of the extracellular signals, long-term studies over days and months are possible [122].

The first application of an MEA chip for the detection of electrically active cells (cardiomyocytes) cultured in vitro was presented in 1972 by Thomas et al. [123]. The MEA used there consisted of gold-plated nickel electrodes that were applied to a glass substrate passivated with a structured photoresist. A glass ring was additionally applied to the substrate around the microelectrodes in order to create a defined culturing area. Since then, microelectrode arrays have been continuously developed. In the first extracellular derivation of dissociated neurons in 1980, Pine [124] used silicon dioxide instead of an organic photoresist to improve the insulation of the conductor paths. The problem of visualizing cells that had grown on the opaque metal microelectrodes could be solved by using transparent electrode arrays made of indium tin oxide (ITO) [125] or conductive organic polymers such as PEDOT:PSS [126]. Despite the worse signal-to-noise (S/N) ratio caused by using the ITO electrodes, Gross et al. used this MEA to stimulate a neural network [127]. In addition to these standard metal electrode MEAs, more complex structures based on open-gate field-effect transistors (FET) were also developed, in which individual neurons were cultured on the gates of the FETs. The first of these MEAs was developed by Fromherz et al. [128] and further developed for the stimulation of cultured neurons [129]. In basic research [130], pharmacology [131], and toxicology [132], commercial MEAs with planar electrode geometry for the use of monolayer cell cultures are widespread.

The proven advantage of 3D over 2D cell culture models is their similar behavior to in vivo behavior when mimicking signaling pathways or drug effects [133,134,135]. This leads to a great interest in the development of 3D MEA systems, addressing the increased complexity of 3D models. Planar MEA electrodes are insufficiently capable of acquiring signals from inside a 3D cell culture model. In order to overcome the limitations of planar 2D electrodes, a large number of different geometries (nanostructures) were tested during the development of 3D electrodes. All of these structures offer the advantage of an enlarged electrode surface and stronger adhesion of the cells to the electrode. Reduced distance between the nanostructured electrode and the cell resulted in improved signal quality [136].

For a spatial resolution of the electrical signals in 3D cell culture models that are several hundred micrometers to a few millimeters in size, needle electrodes with a length of up to several millimeters appear to be suitable. Several microelectrodes can be located on one or both sides on different levels of the electrode shank in order to achieve a spatial resolution of the 3D cell culture model [137]. When using needle electrodes, the 3D cell culture model (e.g., hydrogel) can be built up directly around the electrodes [138], or they can be inserted into the model (hydrogel or spheroid) after the cell culture has matured [139]. This type of 3D MEA also enables optical stimulation of the cells and the introduction of active substances directly into the interior of the 3D cell culture models via hollow needle electrodes [139].

The 3D cell culture models not only enable similar cell behavior to in vivo behavior but also require a more complex nutrient supply due to their size in order to avoid cell death in the center of the culture and to enable long-term cultures. Rowe et al., therefore, developed a scaffold made of a negative photoresist that, in addition, integrates electrodes on different levels of the scaffold, including microfluidic connections for optimal nutrient supply [140]. Another approach to perfused 3D MEA systems was developed by Musick et al., where electroactive cells are cultured on different levels of a cell culture chamber. The levels are micro-fluidically connected to each other and have integrated electrodes. It could be shown that electrical signals could be measured with a high degree of synchronicity between the electrodes of different levels [141].

If dissociated cells, cell aggregates (spheroids), or acute tissue sections are immobilized on planar microelectrodes, the cells adhere through the electrostatic or chemical interactions of the adhesion molecules of the cell membrane with the molecules of the surface coating of the MEA [142]. During cell depolarization and repolarization, the ion currents through the cell membrane cause an electric field in the cell’s proximity, which can be sensed by a microelectrode [143]. The ion current can be altered by influencing the ion channels with suitable inhibitors [144]. To maintain high S/N ratios and space resolutions, the electrode size should not be significantly bigger than the cell size. Because the amplitude extracellular electric field (caused by the action potential) decreases rapidly with the distance from the cell, the recording electrode should be placed within the range of several tens of micrometers. To prevent signal amplitude degradation, the electrode impedance should be as low as possible (electrode impedance is inversely proportional to its surface area), and the input impedance of the recording amplifier should be as high as possible.

#### Design and Construction of 3D MEA Sensors

The 3D MEAs developed by the Institute for Micro and Nano Technologies (IMN) MacroNano^®^ at TU Ilmenau contain needle electrodes for spatial derivation in addition to bottom electrodes. The suitability of these 3D MEAs for measuring neuronal signals has already been proven [145,146].

The MEAs are equipped with a culturing chamber (Figure 8A), which can be perfused with the culture medium. Each 3D MEA has a three-level sensor surface made of gold electrodes to measure cell signals from stacked cell support foils (Figure 8B). The electrodes are arranged as 10 bottom electrodes and 3 needle electrodes, each with 3 electrodes at different heights. The sensing area is surrounded by an integrated reference electrode (Figure 8C).

The measurement setup, described by Bartsch et al. [145], is based on an integrated circuit from Intan Technologies (RHD2132); it enables the simultaneous sampling of all 57 MEA electrodes. Two RHD2132 chips are mounted on a circuit board equipped with spring-loaded contacts for the standard MEA format for 60 electrodes. The circuit board is mounted on an aluminum frame for mechanical robustness and electromagnetic shielding. The structure is controlled via the open-source software RHD2000 (Intan Technologies).

In addition to sensing, automation plays an important role when it comes to efficient drug testing and sampling in an autonomously driven bioreactor.

### 4.4. Automated Culturing and Drug Administration

The acceptance of organotypic 3D models ranges up to millifluidic scale systems [54,55], whereby most systems are based on static, passive fluidics [53,56,57]. However, dynamic (perfused) fluidic systems offer significant advantages, for example, increased transport of nutrients and metabolites within the 3D culture [58,59,60]. The fluid flow also has a direct influence on cell proliferation [61] by virtue of mechanical influences such as shear stress, pressure, and compression, which are essential factors for organ development and function [47]. Advanced cell culture techniques such as microfluidic organs-on-chips (OOCs) offer the opportunity to overcome these limitations. OOC is understood to mean microfluidic systems that are used to culture living cells in micrometer-sized chambers with a continuous flow and to model the physiological and pathological functions of tissues and organs [63]. Regardless of the manufacturing method and the material used, OOCs are suitable for studying biological phenomena that depend on tissue microarchitecture and perfusion [147]. Microfluidic OOCs, therefore, have the potential to become an alternative to animal models [148]. Individual organs-like models of lungs [149], skin [150], liver [151], kidneys [152], heart [153], and gut [154]) and various multi-organ systems [151,155,156] are available. OOCs have also been used for various clinical issues such as drug development and testing [157,158,159,160], cancer therapy [161,162], and oncoimmunology [163] or to identify antiviral therapeutics [164]. 

However, to date, no OOCs have been approved as an in-vitro alternative for animal testing in the relevant guidelines for chemical or pharmaceutical testing [165]. The reason behind this failure is the multiparameter conditioning that must replicate the actual biochemical, geometrical, and fluidic situation in the real organ. Moreover, in perfused long-term cell culture experimental systems, it is particularly important to have an automated medium exchange setup to maintain sterile and reproducible conditions [166]. Additionally, such an automated facility enables the real-time monitoring of cell health [167,168] or functional markers without disassembling the culture system. Since the structure of most body-on-a-chip systems is very complex, online monitoring is suitable for this purpose. Therefore, a number of new generation liver-on-a-chip systems were developed; they are equipped with biosensors or bio imaging to enable the online monitoring of pH and oxygen [169,170] and the cellular metabolic state [171,172] and the detection of cell-derived analytes in the culture medium [120,173,174]. This way, cells do not need to be removed from the perfused culture systems to identify drug toxicity and cellular health. In particular, quantitative analysis of cell-secreted proteins by microfluidic ELISA provides a suitable method for measuring non-invasively the drug toxicity in complex culture systems [120]. However, most studies use PDMS (polydimethylsiloxane)-based chip technologies that strongly interfere with the detection of soluble marker proteins due to adsorption.

**Figure 9:**
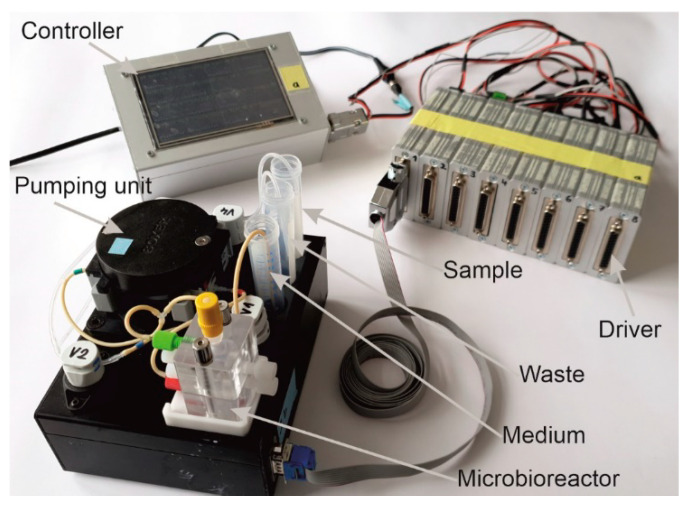
Setup of an automated bioreactor unit for programmable media exchange and sampling.

We have developed a custom-made automated culturing and drug administration device for long-term measurements; it can also be used for the microbioreactors and scaffolds described in this account of research (Figure 9). In principle, the interface is adaptable to any other system with a similar fluidic scale. If the scaffolds are designed in the right way, organ-on-a-chip applications are possible. Because the automated media change is performed in defined periods, the collected medium can easily be fed into the usual laboratory analysis, such as readers, etc.; see the Section 6.

**Figure 10:**
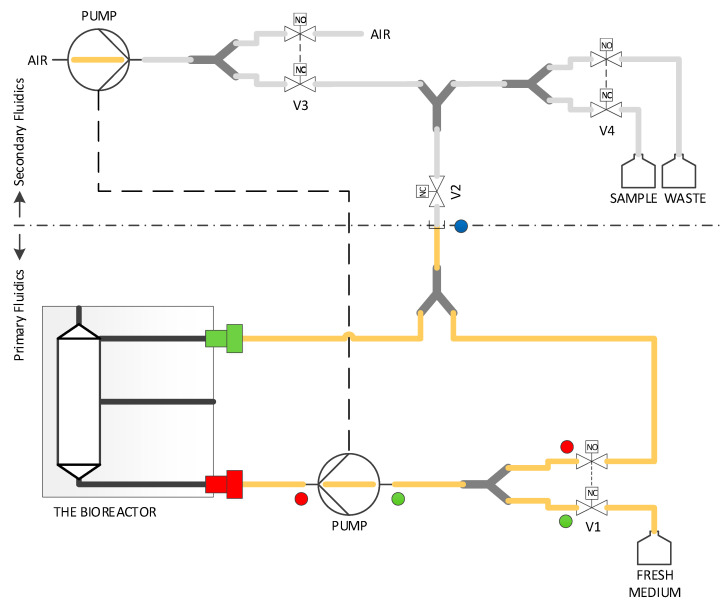
Scheme of the fluidic circuit inside an automated bioreactor unit. For details, see the other study of Baca et al. in this issue.

The perfusion and the automatic medium change are realized via two separate fluidic circuits within the pump and tubing system by switching different solenoid valves. Both circuits run via the same peristaltic pump (Figure 10). The medium in the bioreactor is continuously pumped through the MatriGrid^®^ culture via the primary fluid circuit (perfusion). For this purpose, valve V2 is in the closed state (NC = normally closed), and the fluid circuit runs via the powerless open state (NO = normally open) of valve V1. For the medium change, the valves V1 and V2 are powered and switched on. The medium is now pumped into the secondary fluid circuit via the peristaltic pump and is directed there via the appropriate setting of valve V4 into the sample or waste container. The used medium is, thereby, replaced by the supply of the new medium. After the medium change, the sample in the tubing leading to the waste container is flushed and replaced by air.

An important endpoint in the toxicity assessment of chemicals and pharmaceuticals is repeated administration (repeated dose). The main objective of such an automated fluidic system is to characterize the toxicological profile of the test substance after repeated administration and to identify potential reversibility [175]. In order to replace animal experiments with in vitro methods, culturing systems that allow cells to be cultured in a controlled manner over a long period of time and administer defined repetitive application profiles are required. The presented culturing system with automatic medium change is, therefore, being examined for its suitability as a platform for repeated dose application for long-term experimentation, as discussed in the Section 6.

However, in the heart of every bioreactor are the various culturing scaffolds we call MatriGrid^®^s, which have been manufactured through the micro thermoforming technique described above.

## 5. The MatriGrid^®^-Family

### 5.1. 3D Hepato MatriGrid^®^

**Figure 11:**
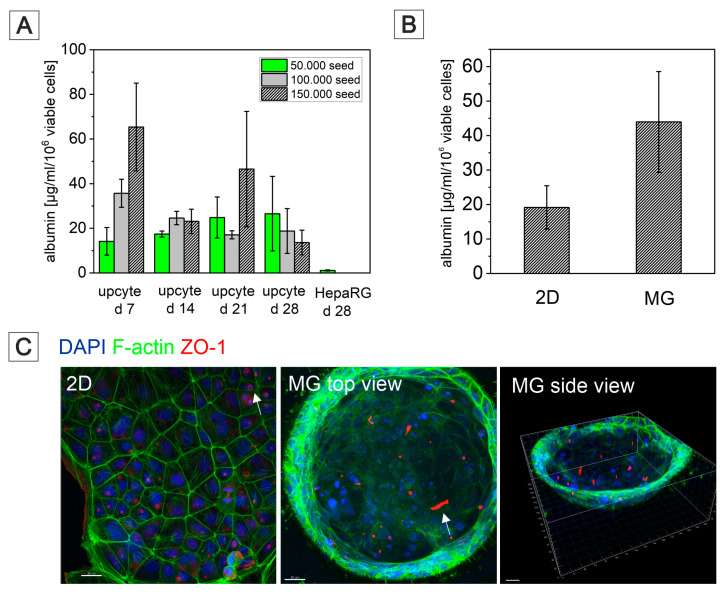
(**A**) Albumin secretion of Upcyte^®^ hepatocytes (donor 422) and differentiated HepaRG cells cultured in MatriGrids^®^ for up to 28 days. Albumin secretion is linked to the cell seeding number. Shown are the mean values ± SD; *n* = 2 experiments. (**B**) Upcyte^®^ hepatocytes (donor 10_03) were cultured for 7 days in 2D or in MatriGrid^®^s, and albumin secretion was measured. Shown are the mean values ± SD; *n* = 3 experiments. (**C**) DAPI/F-Aktin/ZO-1 staining detects *Bile canaliculi* in Upcyte^®^ hepatocytes cultured for 7 days in 2D or in MatriGrid^®^s (MG). Arrows label ring-like *Bile canaliculi* in 2D-cultured cells and tube-like *Bile canaliculi* in MatriGrid^®^s by detection of the tight junctional marker ZO-1. Bars represent 30 µm.

Complimentary to various liver-on-a-chip models [176,177,178], MatriGrid^®^s with typical cavity/container-like morphology were used for culturing human primary Upcyte^®^ hepatocytes and HepaRG hepatocarcinoma cells to mimic organotypic liver growth. Generally, biopsy-derived primary human hepatocytes (PHHs) are the gold standard for in vitro experiments on liver biology and for studying the hepatoxicity of a wide variety of drugs [179]. They can be cultured as monolayers only for a limited period of time due to rapid dedifferentiation and loss of the expression of CYP450 enzymes. Using Upcyte^®^ technology that releases primary hepatocytes from cell cycle arrest by overexpressing the HPV oncogenes E6 and E7 without immortalization, long-proliferating hepatocytes from various donors with stable function were created recently [180]. Many studies have demonstrated that Upcyte^®^ hepatocytes are suitable for preclinical drug metabolism and hepatotoxicity investigations [181,182,183]. Another hepatocarcinoma cell line, the HepaRG cell line, is also convincing due to long-lasting hepatofunctionality, but it has a significant disadvantage, namely, a long differentiation time of over 4 weeks with dimethylsulfoxide [184,185]. Both cell types were established in our MatriGrid^®^ scaffolds and compared in terms of albumin production. Upcyte^®^ hepatocytes were seeded with different starting cell numbers in MatriGrid^®^s, and albumin production was monitored over a time period of 28 days (Figure 11). The highest seed cell number resulted in a sharp increase in albumin secretion after 7 days of culturing in MatriGrid^®^s. Continuous culturing of the cells for up to 28 days resulted in an almost similar and stable albumin secretion that was independent of the seeding cell number. In contrast, differentiated HepaRG cells produced more than 20× less albumin than the Upcyte^®^ hepatocytes grown for 28 days in MatriGrid^®^s.

Additional monitoring of cell numbers and viability revealed gradually increasing cell counts over time and stable viability up to 28 days for Upcyte^®^ hepatocytes (data not shown). MatriGrid^®^ versus monolayer (2D) culturing of Upcyte^®^ hepatocytes for 7 days showed 2-fold increased albumin production due to the 3D environment in MatriGrid^®^ cavities (Figure 11). This impressively demonstrates that the 3D environment provided by the cavity morphology leads to an improvement in the hepatofunctionality of Upcyte^®^ hepatocytes. This result was further supported by labeling *Bile canaliculi* through ZO-1-F-actin staining in monolayer- and MatriGrid^®^-grown Upcyte^®^ hepatocytes. Organotypic hepatocyte culture clearly promotes tubular versus ring-like *Bile canaliculi* labeled by zona occludens protein-1. With these data, we demonstrate that the organotypic 3D culturing of hepatocytes in MatriGrid^®^s significantly improves hepatofunctionality and, thus, is more suitable for studies on liver biology and hepatotoxicity than monolayer culture.

### 5.2. Lung MatriGrid^®^—An Example of Directed Oligocellular Coculture 

Early cell culture models of the lung were mostly based on flat and porous membranes made of, e.g., polycarbonate (PC) or polyethylene terephthalate (PET), on which different combinations of alveolar cells, epithelial cells, and blood cells were cultured [186,187,188,189]. Khalid et al. have developed a lung-cancer-on-chip platform as a promising tool for the cytotoxicity evaluation of novel drug compounds [190].

**Figure 12:**
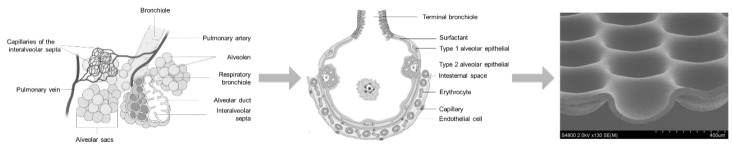
Biotechnical multiscale engineering of the lung.

While using well plate inserts in an air–liquid-interface could be applied to these models to mimic specific lung physiology, Huh et al. [149] were the first to report a lung-on-chip model with a uniaxially stretched membrane to mimic the breathing motion of the lung. In a similar approach, Huang et al. reported a hydrogel-based physiologically relevant model of human pulmonary alveoli [191]. That concept was later improved with a 3D-stained PDMS membrane [192] and the integration of impedance sensors to monitor cell behavior and membrane movement [193]. Furthermore, with respect to the alveoli dimensions, a biodegradable and stretchable biological membrane from collagen and elastin was reported to mimic the central aspects of the air–blood barrier [194]. Comparative to that approach within the MatriGrid^®^-family, a Lung-MatriGrid^®^ based on the shape and size of human lung alveoli was developed using the previously described biotechnical microscale engineering and micro thermoforming technology (Figure 12).

**Figure 13:**
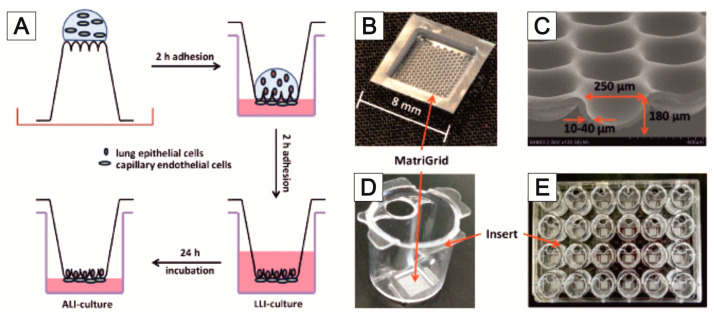
(**A**) Scheme of generation of 3D co-culture. Seeding of endothelial cells on the basal side of the MatriGrid^®^s in a petri dish, transfer to an MTP, and seeding of the apical side with epithelial cells, 24 h incubation as LLI Culture, creation of the ALI culture; (**B**) MatriGrid^®^, (**C**) REM-picture of a sliced MatriGrid^®^, dimensions of the cavities (red arrows), (**D**) semi-active system with MatriGrid^®^ on the bottom, (**E**) 24-well MTP assembled with semi-active systems.

The Lung-MatriGrid^®^ permits the possibility of a 3D oligocellular coculture model of the blood–air barrier with respect to physiological characteristics. The blood–air barrier is characterized by an extremely thin and highly connected layer of epithelial cells that is spread over pulmonary capillaries [195]. Only a 1–2 µm thin structure supports the passive diffusion of respiratory gases [196]. To enable such organotypic exposure of the alveolar epithelia cells against ambient air, the oligocellular coculture model is cultured under an air–liquid interface (ALI) condition. This more physiological ALI culturing is realized by attaching the Lung-MatriGrid^®^ onto the previously described semi-active system (Figure 5). The barrier itself is realized by the seeding of capillary endothelia cells to the basal side of the scaffold, while alveolar epithelia cells are brought into the alveolar-like cavities on the apical side (Figure 13)

**Figure 14:**
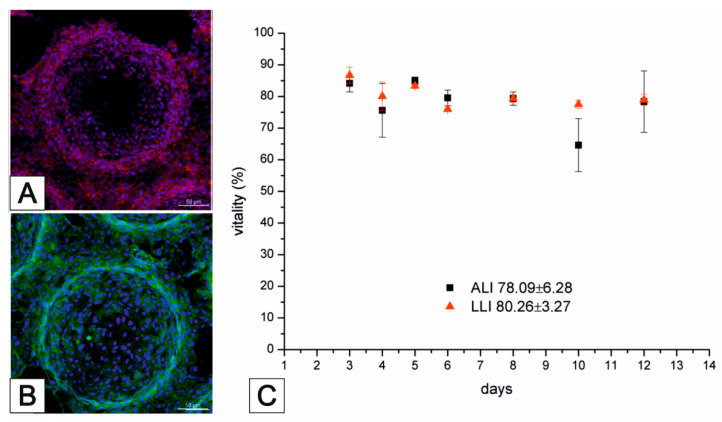
Immunohistochemical stainings of cell-adhesion molecules. (**A**) Zonula occludens-1 and (**B**) E-cadherin; (**C**) comparison of the viability of air–liquid interface (ALI) and liquid–liquid interface (LLI) cultures of A549 on the apical side of the MatriGrid^®^. Bar represents 50 µm.

The medium in ALI culturing is only present underneath the basal side of the scaffold supply of the apical cells, which is ensured by the porosity of the scaffold (pore diameter = 2–4 µm, pore density = 10^6^ pores/cm^2^, thickness = 10–40 µm). Viability and metabolic activity of alveolar epithelial cells (A549) and capillary endothelia cells (EAhy.926) cultured in a lung MatriGrid^®^ are shown to be not compromised in comparison to the culturing in a liquid–liquid interface (LLI) culture over 12 days (Figure 14). Additionally, A549 forms a thick monolayer, with the expression of cell-adhesion molecules (ZO-1, E-cadherin) in the cavities of the Lung-MatriGrid^®^ (Figure 13A). Since the Lung-MatriGrid^®^ can be reversibly separated from the insert system, the cell layer on the scaffold, as well as the cell culture medium, can be evaluated with established methods such as (immuno)-histochemical staining or ELISA to identify changes in cytokine levels or the expression of cell-adhesion molecules due to exposure to, e.g., nanoparticles. Further research is ongoing in the DFG project (DFG 397981139) in cooperation with the Institute of Environmental Toxicology at Martin-Luther-University Halle-Wittenberg to examine the toxicity of BaSO_4_ nanoparticles on primary cells and cell lines in Lung-MatriGrid^®^s. 

### 5.3. NeuroGrid^®^—Scaffolds for the Manipulation and Directed Growth of Neurons and Cerebral Organoids

It is well known that different cellular processes, such as attachment, proliferation, directional migration, and differentiation of neurons, are dependent on morphological and biochemical cues in the surrounding surface [197,198,199]. In this way, the direction and outgrowth of axons and dendrites have been studied by symmetric or asymmetric shapes of trenches [200,201] or specific microplates [202] to guide the connectivity between 3D neuronal cell clusters [203] or to record muscle activity after stimulation of axons in different microfluidic chambers [204]. In addition, there are indications found that the migration capacity of neural cells depends on the stage of neuronal differentiation [205]. Additionally, circular 3D PDMS scaffolds have been used for defining spheroid-like neuronal cell agglomerates [206]. Here, we describe the design of polymeric scaffolds in PC, which should be used as a proof-of-concept study for the handling and shaping of neurons and neuronal organoids. 

**Figure 15:**
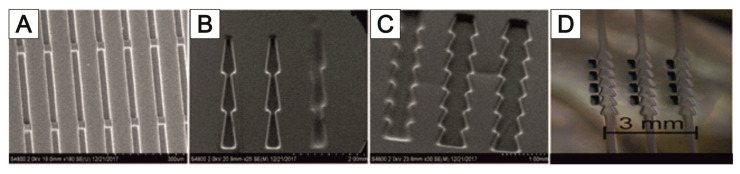
Scaffold designs for guided cell growth; (**A**) vertical and horizontal trenches; (**B**,**C**) different scaled tree structures to mimic the cortical column; (**D**) NeuroGrid^®^ tool with openings for the 3D MEA.

For this reason, it is being investigated whether appropriate modifications of the MatriGrid^®^ scaffold can enable the directed growth of neurons to reproduce desired morphologies, with the vision of developing flexible tools to mimic the complex hierarchies of neuronal tissue. Various designs of MatriGrid^®^s were used to induce the guided growth of embryonic and adult neurons. The set of MatriGrid^®^s with function-dependent embossed structures is given in Figure 15. Besides the fact that neurons are guided easily by microchannels, the geometries of our structures were inspired by the size of neuronal fiber bundles, which are in the range of approximately 500 µm in the case of a cortical column [207,208].

Primary rat cortical neurons were used to allow directed growth through the structures. In the same way, the culturing of neurospheres from induced pluripotent stem cells (iPSC) was investigated. MatriGrid^®^ cultures are also used to bring the guided neurons into close contact with microelectrodes of 2D and 3D MEAs so that it is easier to capture the neuronal signals. That targeted application of MatriGrid^®^ scaffolds can easily be extended by stacking those scaffolds to create complex 3D models to evaluate real 3D network signals from neuronal cells. 

**Figure 16:**
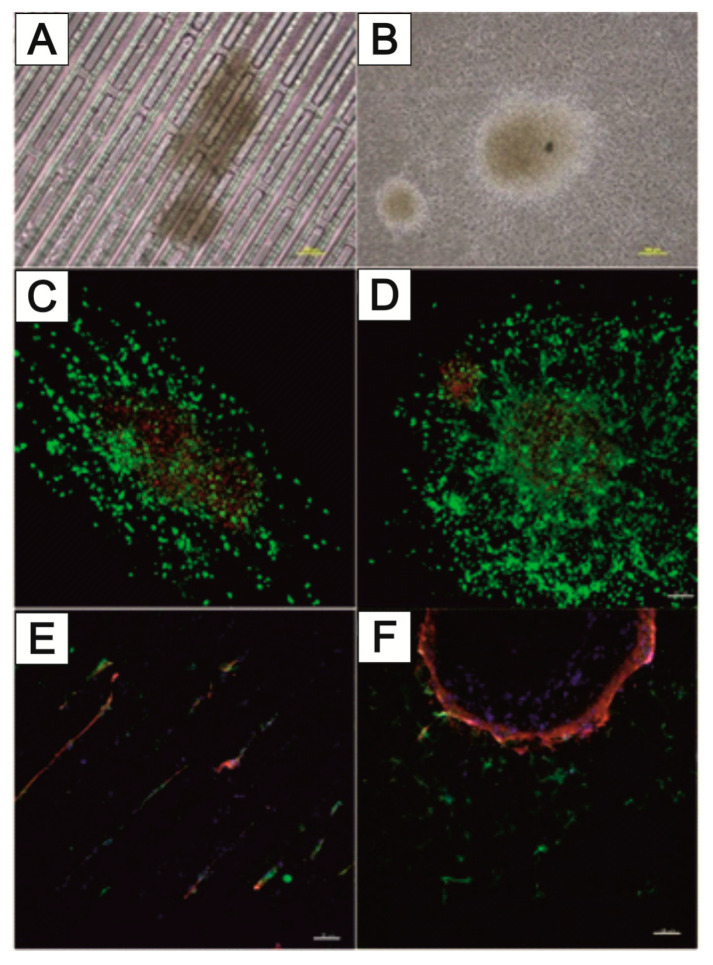
Directional growth of neuronal cells within tranches and radial growth on unstructured PC foil. Brightfield microscopy of the (**A**) channel structure and (**B**) unstructured PC foil. Live–dead staining of neurospheres on the (**C**) channel structure and (**D**) unstructured PC foil (green—viable cells, red—dead cells). Immunofluorescence staining of rat cortex neurons within (**E**) a trench structure and (**F**) on unstructured PC foil (blue—cell nuclei (DAPI), red—βIII-tubulin (TUBB3, Alexa Fluor 594), green—microtubulin-associated protein 2 (MAP2, Alexa Fluor 488)). Bar represents 100 µm.

Figure 16 shows the directional growth of neuronal cells that have grown out of neurospheres. The typical radial outgrowth of neuronal cells from a neurosphere can be seen on the unstructured PC foil (Figure 16B), although it is not possible to evaluate the growth of neurons within the trench structure using bright field microscopy (Figure 16A). Cell staining is needed to estimate any guided neuronal cell growth inside the narrow NeuroGrid^®^-structures. Live–dead staining as well as immunofluorescence staining against neuronal markers microtubulin-associated protein 2 (MAP2) and βIII-tubulin (TUBB3) were performed. In addition, the cell nuclei were stained with DAPI (blue). The trench structure forces a directional growth compared to the unstructured PC foil (Figure 16C,D). Mainly viable (green-stained) cells outside the neurospheres can be seen. Viable cells, on the other hand, are present in the neurospheres. While TUBB3-stained neurons grow along the trenches, MAP2-stained cells do not undergo this directed growth and are also found in the areas between the trenches (Figure 16E). In contrast, on the unstructured PC foil, mainly MAP2-stained cells grow out of the aggregates of the rat cortex neurons, and only a small number of TUBB3-stained cells can be seen (Figure 16F). 

**Figure 17:**
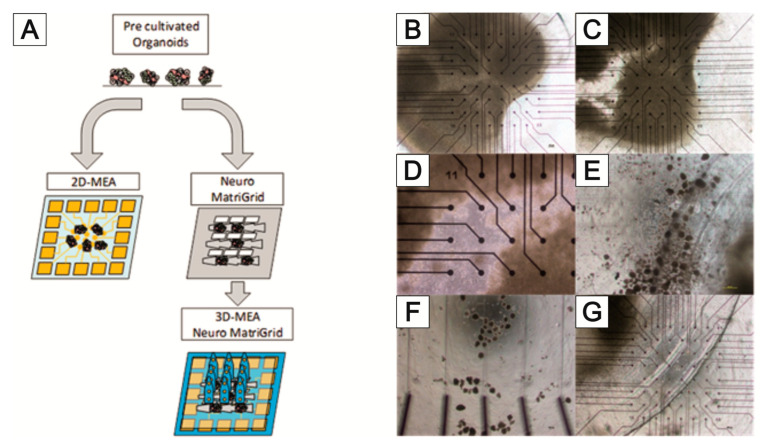
(**A**) Principles of targeted application of neuronal cells by MatriGrid^®^; (**B**) neurospheres transferred from MatriGrid^®^ to 2D MEA after 1 day; (**C**) 6 days and (**D**) 9 days of culturing; detached neuronal cells (**E**) at the edge of the applied foil and (**F**) on the outer MEA surface; (**G**) PC film slipped after medium change.

A major problem in deriving neuronal signals is the growth of neuronal cells outside the sensor area of the MEA. This circumstance makes planning more difficult and prevents the standardization of the experiments. For this reason, the targeted application of neuronal cells to the electrodes of the 2D and 3D MEAs was tested using MatriGrid^®^ foils (Figure 17A). Normal PC foils and MatriGrid^®^ with a towering tree structure were used for the experiments with 2D MEAs. Special 3D MEA foils with cutouts for the 3D needle electrodes were used for targeted positioning on 3D MEAs. In both variants, neurospheres were pre-cultured on the foils for 7 days and then transferred to the 2D or 3D MEA and cultured further for at least 7 days. The signals were recorded daily after the transfer to the MEA. The neurospheres for the 2D MEA were pipetted as centrally as possible onto the foil or into the tree structures of the MatriGrid^®^. For the 3D MEA, neurospheres were placed in the structures on the ridges between the cutouts for the needle electrodes. All foils were coated with Geltrex in order to cover the entire foil with cells. Figure 17 shows the behavior of the neuronal cells after application over a culturing period of 9 days. While the neurospheres are intact on the first day after the transfer (Figure 17B), they show gaps during longer culturing, which become larger the longer the foils are cultured on the MEAs (Figure 17C,D). Individual outgrown neuronal cells can be seen within these gaps. However, it cannot be seen whether these have grown on the foil or the MEA (Figure 17D). After a few days, detached cells are present in the medium, both at the edge of the foils placed (Figure 17E) and over the remaining MEA surface (Figure 17F). These do not adhere to the surface of the MEA but form cell aggregates. It could also be observed that the PC film slips on the 2D MEA when changing the medium (Figure 17F).

**Figure 18:**
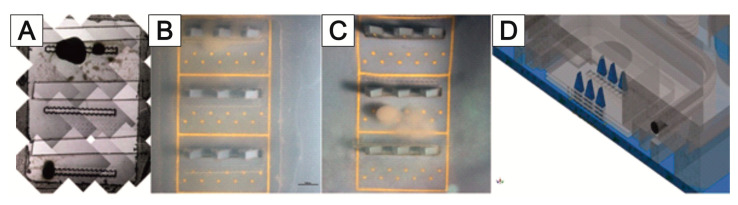
The 3D MEA foil for the targeted application of neurospheres on MEA electrodes. (**A**) Bright-field image of the 3D MEA foil before transfer to the 3D MEA; (**B**) 3D MEA without 3D MEA foil; (**C**) 3D MEA with 3D MEA foil and spheroid with contact with the needle electrodes; (**D**) sketch of a 3D MEA with bioreactor housing and 3D-stacked NeuroGrids; bar represents 500 µm.

Figure 18 shows the 3D MEA foil before it was transferred to the 3D MEA (Figure 18A). When the foil was applied, it was transferred to the 3D MEA with the cells facing down. When neurospheres are cultured in the tree structures of the 3D MEA foil, they can be placed directly on the needle electrodes (Figure 18B); when cultured on the ridges between the cutouts, it is also possible to position the neurospheres specifically on the bottom electrodes of the 3D MEA (Figure 18C). It should be noted that individual neurospheres can become detached from the 3D MEA film during transfer (comparison of Figure 18A,B).

**Figure 19:**
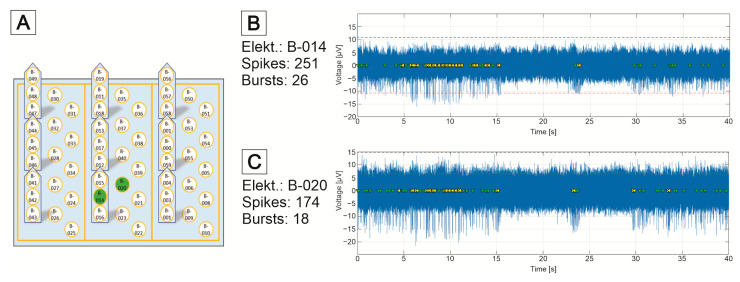
Neuronal signals measured after transferring the 3D MEA film to a 3D MEA (7 days of pre-culturing in a 6-well MTP and 7 days of culturing on the MEA) (**A**) Electrode array with numbering of the electrodes (**B**) Measurement from middle of the needle electrode (B-014) (**C**) Measurement on bottom electrode (B-020).

Figure 19 shows an example of a 3D MEA measurement of neuronal signals after the targeted application of a 3D MEA foil. Neural signals were measured at the opposing electrodes B-014 and B-020 (comparison of Figure 19A,B). Here, electrode B-020 is a bottom electrode that is opposite the middle needle electrode B-014. The signals from the two electrodes show a high degree of synchronicity (Figure 19C). The background noise of the electrodes is between 5 and 10 µV; mainly negative spikes with a maximum of 20 µV were measured. Both signals contained bursts (see Materials and Methods in the Supplementary Information).

**Figure 20:**
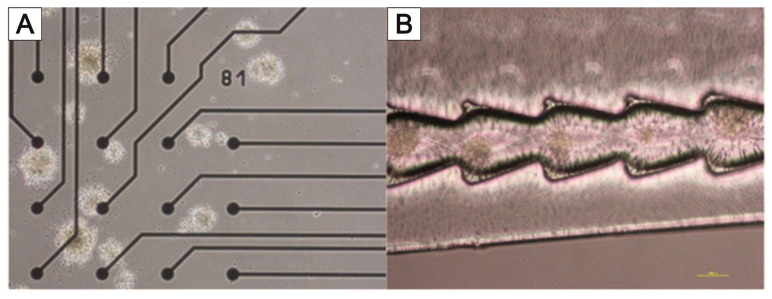
(**A**) Spheroids generated from cortex neurons in MatriGrid® adhered to 2D MEAs and (**B**) in the fir-tree structures of the 3D MEA foil. Bar represents 100 µm.

Besides the use for the directed growth of neurons and a targeted application to 2D or 3D MEAs, the MatriGrid^®^s can also be used as a handling tool to create more uniform spheroids of neuronal cells. For this purpose, dissociated rat cortex neurons were pipetted into the cavities of the MatriGrid^®^s. The cavities were previously coated with anti-adherence rinsing solutions. The spheroids precultured and shaped in the MatriGrid^®^ were transferred directly onto 2D MEAs or into structures of other MatriGrid^®^s (Figure 20). For both approaches, the attachment of the spheroids and the outgrowth of neurons were verifiable. A big advantage of that approach in forming spheroids is the possibility of defining the diameter of the spheroid through the size of the cavities. Because of that, the spheroids are highly adaptable to the purpose they will be used for.

### 5.4. TissGrid^®^

**Figure 21:**
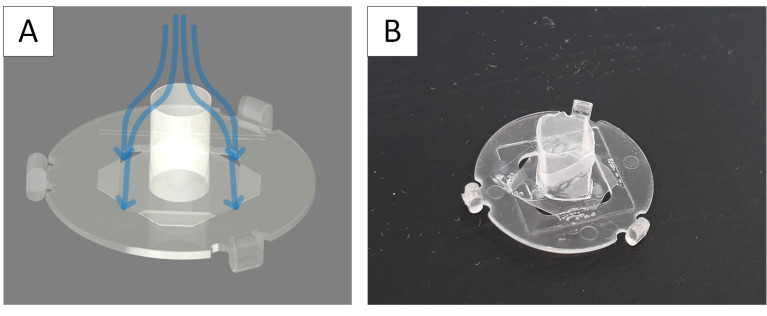
(**A**) Schematic of TissGrid^®^ with the flow path of the fluid; (**B**) the manufactured TissGrid^®^.

The need for adapted scaffold structures is not only important in cell cultures based on cell lines or primary cell cultures; the culturing of explants or tissue slices can also benefit from the advantages of a scaffold approach. The scaffold creates an adapted microenvironment for the specific explant and, thus, optimal survival conditions. This is particularly important for longer culturing periods. Flow-induced shear stress has a big influence on cells and tissues [209,210,211,212,213], either in a positive way, mimicking the effects of vascularization, or in a negative way on sensitive cells and tissue slices, where the stress damages the cells. In a case study, we have examined this effect on placenta tissues, called placenta explants, because drug and particle transport across the human placenta is a deciding factor for fetus development [214,215]. It is generally known that fluidics also have a major influence on the cellular phenotypes of the placenta [216]. Therefore, it is desirable not only to culture the explants statically but also to culture them inside microfluidic systems. Placenta explants are sensitive tissue structures that lose their integrity under fluid shear stress; they cannot maintain their viability for an indefinite period. We examined the fluid stress effects on explants under different fluidic regimes. Following the observation that placenta explants are very sensitive, we designed a new scaffold structure that combined the potential shelter function of a porous cavity with the advantage of better fluidic supply with respect to nutritious flow. The TissGrid^®^ structure is designed in the following way. A central cylindrical cavity standing on a porous base surface is used to accommodate the explant. This can be easily inserted into the scaffold from above without damage. The cavity is made of microporous transparent polycarbonate film by thermoforming. Microscopic observation of the explant is possible during culturing. Due to the porosity, a very good diffusive supply of cells is possible. In order to achieve an effective flow around the explant cylinder and, thus, high diffusion gradients, bypass openings were inserted at the corners of the base surface. The base surface was also made of porous polycarbonate film, which also allows the microscopic inspection of the samples. To integrate the system into the microreactors described above, the scaffolds were fixed onto a carrier chip. In order to avoid an undesired influence on cell culture, no adhesives should be used to bind the scaffolds. Especially in long-term cultures, substances may leach out of the adhesive. In the application described here, the scaffold parts were, therefore, bonded with solvent. This could be completely removed by appropriate heat treatment in a vacuum. This way, easy storage and handling and good sealing of the scaffold in the bioreactor are possible. A schematic representation of the TissGrid^®^ is shown in Figure 21.

We were able to show that with the specially designed TissGrid^®^s, a flow-through protective structure could be set up, which enables the explants to be supplied with medium/serum flowing past while maintaining viability [217].

**Figure 22:**
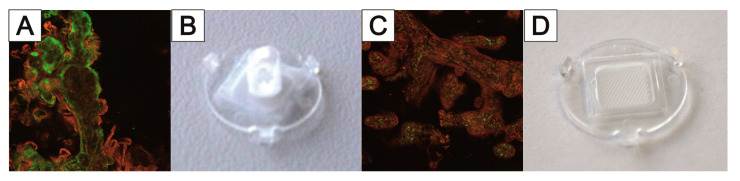
Live–dead assays to determine the viability of placental explants after 14 days of culture under fluidic conditions are shown to the left of the TissGrid^®^ (**B**) and the MatriGrid^®^ (**D**). Viable tissue is green; dead tissue is red. The villous structures of the placenta react very sensitively to shearing forces, which leads to a significant reduction in the viability of the placental tissue in the MatriGrid^®^ (**C**). The protected environment in the TissGrid^®^, on the other hand, enables excellent regeneration of the placenta explant (**A**). In the live–dead assay, the original degenerated syncytiotrophoblast (red) can be seen, which has been replaced by a newly formed one (green) on the surface of the explant (**A**).

Under conventional culture conditions, without the influence of test substances, we were able to observe relatively stable glucose consumption and stable lactate production in placenta explants in a conventional microtiter plate (MTP) for up to 10 days, which indicates good placental functionality and metabolism. By changing the culture conditions with the help of specially developed fluidic systems (TissGrid^®^s in bioreactors [217]; Figure 22), a placental-active metabolism could even be stimulated, which shows an increased production of estradiol by the syncytiotrophoblast at flow rates of 100 µL/min of the culture medium (unpublished data). The substrate structures were thoroughly tested and led to the specially developed TissGrid^®^. In addition, different flow rates were varied in these experiments.

In contrast, the relative decrease in estradiol production under static (not perfused) conditions (2D, static plate) and the low flow rates of 10 µL/min indicate the degeneration and decreased function of the explants. Preliminary tests also showed that it was not possible to carry out tests at high flow rates in the structures developed for 3D cell culture (called MatriGrid^®^s (Figure 22)). The tissue lost its intact structure (Figure 22C). With the help of the TissGrid^®^s in special MTPs or microbioreactors designed for this purpose (Figure 22A), which are to be standardized, a further time window can be opened up due to the supply of the placenta explants with culture medium.

Besides the culturing of primary cells and even explants in fluidic setups, as described before, long-term experiments bear a high potential for the application of MatriGrid^®^ scaffolds. In the following section, we describe a selected example where hepatocytes were cultured and characterized for up to 28 days.

## 6. Long-Term Automated Culturing and Drug Administration

For the examination of hepatocytes after repeated administration of a hepatotoxic substance, primary Upcyte^®^ hepatocytes (uHep) were seeded in the MatriGrid^®^.

Upcyte^®^ hepatocytes (uHep) are established as a model for the metabolism of metabolically stable compounds [218]. Primary donor cells are used for uHeps, which are stimulated to proliferate using the E6 and E7 genes of human papilloma virus 16 [180]. This has the advantage that the immortalization avoids a change in the phenotype of the primary liver cells. The activities of CYP1A2, CYP2C9, and CYP3A4, when stimulated by CYP inhibitors, are equivalent to or higher than their activities in primary human hepatocytes [219], but fluctuations in the expression of various genes between the individual donors can be observed [182,219,220].

**Figure 23:**
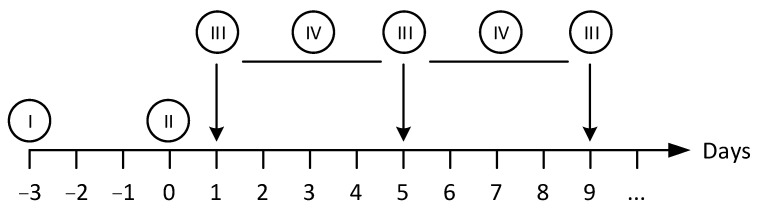
Schematic test procedure for repeated addition of acetaminophen. (I) Sowing in MatriGrid^®^ and static “pre-culturing” in MTP for 3 days; (II) installation in microbioreactors and culturing, including daily medium change in the automatic culturing system; (III) addition of the drug acetaminophen; (IV) incubation for 4 days.

The schematic test procedure is shown in Figure 23. For the test, 150,000 uHeps of donor 10-03 was used as the starting cell count and “pre-cultured” statically in the MatriGrid^®^ in an MTP for 3 days (Figure 23I). The MatriGrid^®^s were then transferred to the bioreactors (Figure 23II), where they were cultured automatically. The MatriGrid^®^ cultures were perfused at 12 µL/min, and the medium was changed daily. The first addition of the drug acetaminophen (APAP) took place after 24 h during the medium change and then again after 4 days of incubation (Figure 23III). The drug addition was repeated six times. Between drug additions, the cell cultures were incubated with daily medium changes (Figure 23IV). The exchanged medium was collected separately for each bioreactor. APAP concentrations of 0.25, 2, and 5 mM were investigated. For this purpose, the albumin and LDH concentrations were determined from the collected medium.

**Figure 24:**
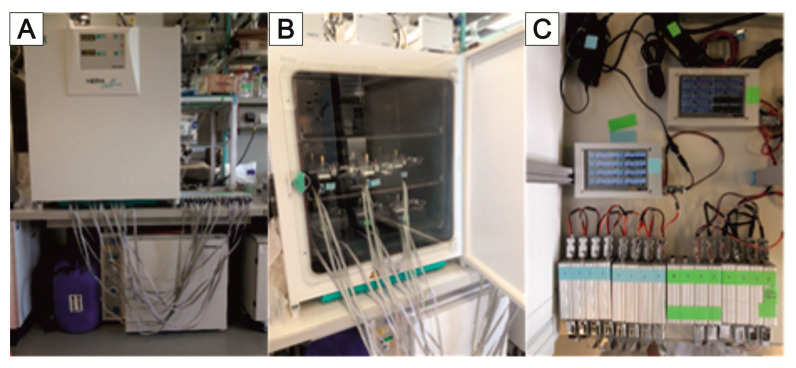
(**A**) Laboratory set-up for the repeated addition of active ingredients with automatic culturing systems; (**B**) color-coded and numbered culturing systems in the incubator; (**C**) control units for the culturing systems used in parallel.

Figure 24 shows the laboratory setup for the experiment. Here, 14 parallel operating bioreactors (2× control, 4 × 0.25 mM, 4 × 2 mM, and 4 × 5 mM) were kept in the incubator throughout the experiment. 

To investigate the suitability of the automatic perfusion system for repeated dose studies, the repeated addition of acetaminophen in concentrations of 0.25, 2, and 5 mM was tested. The long-term culturing of the primary Upcyte^®^ hepatocytes (uHep) in the bioreactor with automatic medium change by the culturing system was carried out as mentioned above. Albumin secretion and LDH leakage by the Upcyte^®^ cells, treated with repeated doses of APAP, were normalized against the initial values measured on the first day of culturing, which were set to 1.

**Figure 25:**
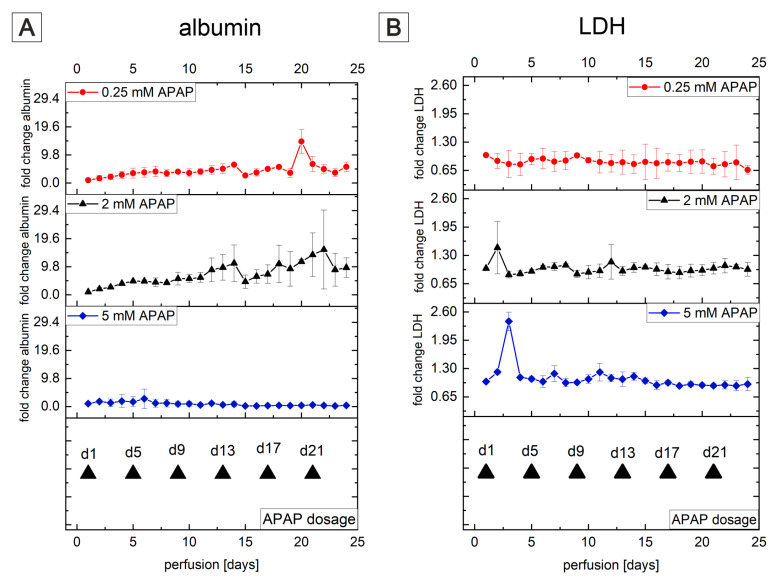
Repeated APAP applications of 0.25, 2, and 5 mM on uHep MatriGrid^®^ culture during perfusion in the bioreactor. (**A**) Fold change in albumin secretion; (**B**) LDH leakage in the cell culture medium during perfusion culturing. The timepoint of APAP dosage is marked with black triangles. Following APAP applications, the cells were perfused with fresh medium to allow cell recovery. Shown are the mean values ± SD; *n* = 1 experiment for two bioreactors for each concentration.

Figure 25A shows the course of albumin secretion from the Upcyte^®^ hepatocytes after repeated administration of 0.25, 2, and 5 mM APAP. Relative to albumin secretion on day 1, the addition of acetaminophen at the concentration of 0.25 mM resulted in a continuously increasing albumin secretion of the treated Upcyte^®^ hepatocytes, peaking on day 20 with a 14.5-fold increase. Repeated addition of 2 mM APAP also resulted in an increase in albumin synthesis, with two peaks on day 14 (12-fold increase) and day 22 (16-fold increase). It was observed that each addition of APAP led to a short impairment of cellular albumin secretion, followed by a further enhancement in albumin synthesis to an even higher level. This effect was most notable at a concentration of 2 mM APAP. Repeated dosage of a high APAP concentration (5 mM) impaired albumin secretion by the Upcyte^®^ hepatocytes from the beginning, and after the APAP pulse on day 5, the cells almost ceased albumin secretion.

Analysis of LDH secretion (Figure 25B) showed that after the first pulse of the highest concentration of APAP (5 mM), cell death occurred, visible as a 2.6-fold increase of LDH secretion on day 3 of perfusion culturing. A slight increase in LDH release was also observed after the first dose of 2 mM APAP on day 2. The lowest APAP concentration (0.25 mM) did not lead to significant LDH leakage, indicating that no cell damage occurred at this APAP concentration. 

We were able to perfuse Upcyte^®^ hepatocytes seeded in MatriGrid^®^s in an automated perfusion system for up to 24 days. Relevant biological endpoints (albumin, LDH) were used for the analysis of the cell health and functionality of the perfused Upcyte^®^ hepatocytes. A repeated dose–response study, with APAP as the investigated drug, showed the reversible induction of albumin synthesis by APAP concentrations of 0.25 and 2 mM. An up-to-16-fold increase of albumin production by 2 mM APAP, compared to the albumin secretion at the first perfusion day, was detected. It is conceivable that under stress conditions (in the presence of the toxic APAP), the cells increase their metabolism to reduce toxicity. The increase in albumin synthesis could have a counter-regulatory function in binding the high drug concentrations in the blood. In contrast, a higher APAP concentration (5 mM) impairs the albumin production of Upcyte^®^ cells directly after the first drug addition, with no apparent recovery over time. This indicates cellular damage leading to decreased cell functionality, as shown by reduced albumin secretion. In experiments with spheroids from HepG2/C3A cells [221], Fey et al. found a chronic impairment of viability of the cells to the repeated addition of 10 mg APAP per mg of protein. Lower concentrations showed no lethal effect, while higher concentrations led to an immediate loss of cell viability. In studies with a significantly higher APAP concentration (18.6 mM) on primary hepatocytes in 2D cultures in 96-well MTPs, a reversible decrease in albumin concentration after the addition of the used drug was also observed [222,223].

## 7. Conclusions and Outlook

We close the description of our account of research with a classification with respect to contextualization and a discussion of further perspectives of technological development.

### 7.1. Conclusions

We have shown that polymeric substrates for 3D culturing can be adapted to the needs of specific cells of different organs. Starting with the basic idea of forcing cells to develop 3D contacts with each other by simultaneously guaranteeing a sufficient supply of oxygen and nutrients [92,224], the first MatriGrid^®^ was developed with defined porous and non-porous regions in its structure. We apply this basic structure to different liver cell types. Such cavities reflect even the real morphology of lung alveoli, which opens the way to an oligocellular model of the alveoli, with site-specific adhesion of the suitable cell types. Such MatriGrid^®^s can be integrated into insert systems that are used in MTPs or into our own specially developed micro bioreactors. Other morphologies, such as linear trenches, are suitable for neurons and can be used for the examination of the differentiation and evolution of neuronal cells. In addition, such cavities also allow us to form organoid-like structures. Furthermore, these NeuroGrid^®^s can be used for the transfer and manipulations of organoids for analytical purposes. A family of micro bioreactors that are optimized for our MatriGrid^®^s has been established, including a system for automatic long-term culturing and drug administration, allowing for experiments of even one month. 

Specially adapted TissGrid^®^ structures with an optimal fluidic design have also opened a way for culturing tissue slices, explants, and biopsy material in bioreactor devices. A short overview of the pros and cons of the presented systems is given in Table 2.

### 7.2. Contextualization and a Discussion of Further Perspectives of the Technological Development

We have chosen template transfer technology based on the principles of biotechnical multiscale engineering as the main strategy of designing scaffolds for advanced cell culture. Within this frame, microforming technologies are a versatile tool to construct cost-efficient microfluidic substrates, especially for cell culturing. Nevertheless, there are drawbacks that have to be considered. One issue is the design of real free-form 3D lumen-like structures, which might also be problematic for all traditional microsystem technologies. Using connection and packaging methods, such 3D lumen structures are realized, which is also possible for thermoformed foil designs. Within this scope is the clapping and stacking technology developed by our department [225]. Another way forward might be a family of 3D printing technologies, which is promising but still far from high-throughput production in this field.

Additionally, it would be fruitful to combine microforming with traditional micromachining technologies; one solution could be advanced lamination technologies such as thermo-blow mold lamination [226], which would allow for the integration of sensors and actuators in such micro devices in combination with polymer foil technology.

Within this context, especially for the examination of electroactive cells, it would be fruitful to integrate electrodes in polymeric fluidic and even cell culture substrates. One solution could be PEDOT-based polymeric systems [126]; however, these approaches lack the possibility of combining electronics in such a way that even porous substrates can be combined with fluidics.

Guided site-specific positioning and adhesion of specific cell types remain challenging, but it is a necessity for the future design of oligocellular organotypic devices. We have shown some building blocks on the way to this future, but there is still no general way to the complete realization of this promising scenario. Furthermore, the combination of the methods described above should allow the design of hybrid systems that mimic liver lobules or the cortical column. Nevertheless, the interplay between shape and morphological development, mechanical and physical cues, and cell differentiation gives a strong hint for the further development of scaffold-based cell culturing devices for the reproducible culturing of organoids, which should help pharmaceutical drug testing and the avoidance of animal testing especially. 

Routine applications of automated systems for drug administration have demonstrated robust behavior, but the simultaneous use of more than 16 reactors, together with their driver units, in one regularly sized incubator is challenging. For this purpose, the parallelization and miniaturization of micro bioreactors would be advantageous. Other groups [68,227,228,229] have also recognized the need for parallelization and miniaturization, which will gradually become more and more important. For this reason, we are developing a parallel micro bioreactor platform based on a 24-well plate. Each well receives a micro pump so that active perfusion of the MatriGrid^®^s can take place independently in each well. The basis of the system is a 24-well from Greiner, into which the MatriGrid^®^s are inserted; the pump plate is then placed. In addition, this system is open on an ordinary 24-well plate so that the medium can be removed or added at any time with a pipette.

## Figures and Tables

**Table 1:** Comparative account of selected liver models based on our interpretation of structural and practical considerations.

Method	Multi-Cellularity	Physiological Micro-Circulation	Lobule-Mimetic Cell Pattern	3D Architecture	Throughput	Imaging	Unique Benefits (✔) and Potential Limitations (✖)
Lobule-mimetic DEP cell patterning [64]	●	-	●●	-	-	●●	✔ very well-controlled cell–cell interactions ✔ compatible with high-content imaging readouts ✖ requires specialized equipment and devices
Cell sheet engineering technology [65,66,67]	●	-	●●	●	-	-	✔ great potential to fabricate unique, functional cell-dense tissue constructs ✖ problems including hypoxia, nutrient insufficiency, and waste accumulation may occur ✖ fragile and difficult to handle ✖ maximal thickness of the construct is limited
Scaffold based bioreactor [68,69,70]	●	●	-	●●	●●	●	✔ ease of handling, applicable to microplates ✔ ability for in situ microscopic examination ✖ spherical morphology of the cultured cells may cause difficulties in oxygen and nutrient diffusion ✖ of spatial distribution of co-cultured cells
Scaffold-free spheroids in perfused stirred-tank bioreactors [71]	●	●	-	●●	●●	-	✔ formation of 3D cellular aggregates in a controlled size ✔ spatial segregation of the co-cultured cells ✖ difficulties in high-content imaging for entire spheroid ✖ difficulties in oxygen and nutrient diffusion through large aggregates (spheroids size limitation ~200 µm) ✖ can be difficult to control disorganized cell type interactions over time
Liver-on-a-chip platforms based on layer-by-layer cell deposition on microporous membranes [72,73]	●●	●	●	●	-	●●	✔ easy to control the position of cell layers to mimic the distribution of liver cells ✔ utilizes suspended membranes as cell substrates, mimicking the space of Dissé ✖ non-specific binding of drugs to chip materials ✖ applicable to small volumes of cells ✖ shear stress may cause lower hepatic functions
Hollow-fiber bioreactor [74]	●●	●●	●	●	●	●	✔ unique fluid flow, mimicking capillary blood–tissue exchange ✔ the fiber shields hepatocytes from the shear stress associated with perfusion ✖ complex system, difficult to establish ✖ binding of drugs to scaffold
Bundling-up assembly of cell-laden hydrogel microfibers [75,76]	●	●	●	●	-	-	✔ allows encapsulation of diverse cells in a controlled environment ✔ mimics hepatic cord structures ✔ fiber shape enables a good exchange of nutrients and oxygen ✖ complex system, difficult to establish ✖ requires specialized equipment and devices
Multicellular hierarchical micromodules fabricated using shape-controllable photolithography [77,78]	●	●	●	●	●	-	✔ micromodules could be spatially organized, layer-by-layer, to form a 3D construct ✔ enables the creation of vessel-like lumen ✖ UV irradiation can influence cells ✖ requires specialized equipment and devices
Bioprinted liver organoids [79,80,81,82]	●●	-	●●	●●	-	-	✔ precise control of cell placement ✔ allows creation of diverse architectures as desired ✖ requires complex and expensive equipment ✖ potential heterogeneous nutrient or drug distribution within large and cell-dense bioprinted tissues ✖ vascular network has not been fully developed, with only a few exceptions [80] ✖ high sheer stress to the cells during fabrication
Decellularized human liver repopulated with cells [83]	-	-	●●	●●	-	-	✔ extremely well-preserved 3D microanatomy of the liver lobules ✔ expression and distribution of key ECM components of the liver tissue are fully maintained ✖ requires a long decellularization process ✖ very difficult to uniformly introduce cells or target different types of cells to their correct location ✖ potential xenogenic immune problems

Double dot (●●) means excellent replication or performance, single dot (●) means partial replication or performance, and minus (-) means the absence of the desired characteristics.

**Table 2:** Summarized pros and cons of MatriGrid^®^-based scaffolds in technical terms.

System	Pros	Cons
Hepato-MatriGrid^®^ Lung-MatriGrid^®^ NeuroGrid^®^	Easy to manufacture in an appropriate quantity	Difficult microscopic observation through the 3D structure Currently no active membrane elements
	Straightforward production of 3D cell cultures	Selection of available materials is limited
	Fits in a 24-well plate	
	Can be used in a bioreactor as well as in a well plate	
	Pores and channels can be site-specific-modified	
TissGrid^®^	Fits in a 24-well plate	Currently producible only in small quantities
	Can be used in a bioreactor as well as in a well plate	Selection of available materials is limited
	Low shear stress on the cells at high flow rates	
3D MEA System	3D electrode array	Not transparent
	can be used together with the NeuroGrid^®^	
Microbioreactor	Easy to use	Complicated for more than 8 units
	Flexible, different flow conditions can be realized	In most cases, an external pump is necessary
	The bioreactors can be connected in a serial way	
	Even dilution of metabolic products is supported	
Microbioreactor unit	Automatic medium exchange	Relatively large footprint
	Automatic sample drawing	
	Automatic long-term culturing	
	Up to 8 systems can be managed by one controller	

## Data Availability

No additional data, accept for the Supplementary Material is provided.

## Supplementary Materials

The following supporting information can be downloaded at: https://www.mdpi.com/article/10.3390/bioengineering9050220/s1, Selected information regarding the micro bioreactor, MatriGrid^®^ production, cell culture, staining methods, and MEA measurements can be found in the Supplementary Information material and methods [119,230,231,232,233,234,235].
