# Supplementary material for: MatriGrid® Based Biological Morphologies: Tools for 3D Cell Culturing
Source: Bioengineering (Basel). 2022 May 20;9(5):220. doi: 10.3390/bioengineering9050220 (PMC9138054; doi:10.3390/bioengineering9050220)
Supplement: Supplementary file 1 [file bioengineering-09-00220-s001.zip › bioengineering-1696634-supplementary.pdf]

bioengineering

MDPI

-supplementary information-

MatriGrid® Based Biological Morphologies: Tools for 3D Cell Culturing

For special issue: Analytical approaches in 3D in vitro systems

Patrick Mai [1,†,‡], Jörg Hampl [1,*,†], Martin Baca [1,†,‡], Dana Brauer [1], Sukhdeep Singh [1], Frank Weise [1], Justyna Borowiec [1], André Schmidt [2], Merle-Johanna Küstner [1], Maren Klett [1], Michael Gebinoga [1], Insa S. Schroeder [3], Udo R. Markert [2], Felix Glahn [4], Berit Schumann [4], Diana Eckstein [4] and Andreas Schober [1,*]

[1] Department of Nano-Biosystems Engineering, Institute of Chemistry and Biotechnology, Ilmenau University of Technology,98693 Ilmenau, Germany; maipatrick@outlook.de (P.M.); martin.baca@emse.fr (M.B.); dana.brauer@tu-ilmenau.de (D.B.); sukhdeep.singh@tu-ilmenau.de (S.S.); frank.weise@tu-ilmenau.de (F.W.); justyna.borowiec@tu-ilmenau.de (J.B.); merle-johanna.kuestner@tu-ilmenau.de (M.J.K.); maren.klett@tu-ilmenau.de (M.K.); michael.gebinoga@tu-ilmenau.de (M.G.)

[2] Placenta Lab, Department of Obstetrics, Jena University Hospital, 07747 Jena, Germany; andre.schmidt@med.uni-jena.de (A.Schm.); markert@med.uni-jena.de (U.R.M.)

[3] GSI Helmholtzzentrum für Schwerionenforschung, Biophysics Division, 64291 Darmstadt, Germany; i.schroeder@gsi.de (I.S.)

[4] Institute of Environmental Toxicology, Martin-Luther-University Halle-Wittenberg, 06097 Halle/Saale, Germany; felix.glahn@uk-halle.de (F.G); berit.schumann@uk-halle.de (B.S.); diana.eckstein@uk-halle.de (D.E.)

* Correspondence: joerg.hampl@tu-ilmenau.de (J.H.); andreas.schober@tu-ilmenau.de (A.S.); Tel.: +49-3677-6933387 (A.S.)

† These authors contributed equally to this work.

‡ former coworker, current affiliation: (P.M.) Lonza AG, Quality Control Microbiology—Cleaning Validation, 3930 Visp, Switzerland

‡ former coworker, current affiliation: (M.B.) MINES Saint-Étienne, Department of Bioelectronics (BEL), 42023 Saint-Étienne Cedex 2, France.

Citation: Mai, P.; Hampl, J.; Baca, M.; et al. MatriGrid® Based Biological Morphologies: Tools for 3D Cell Culturing. *Bioengineering* **2022**, *9*, x. https://doi.org/10.3390/xxxxx

Academic Editor(s): Boštjan Vihar

Received: 08 April 2022
Accepted: 11 May 2022
Published:

Publisher's Note: MDPI stays neutral with regard to jurisdictional claims in published maps and institutional affiliations.

Copyright: © 2022 by the authors. Submitted for possible open access publication under the terms and conditions of the Creative Commons Attribution (CC BY) license (https://creativecommons.org/licenses/by/4.0/).

1. Description of selected processes used for culturing cells in different MatriGrid®s and methods thereof

1.1. O_2 Consumption of a liver lobule theoretically

The liver has a weight of 1.5 kg and supplied with an average of approximately 1.5 l/min blood. One third of this blood flow is arterial blood [1]. The oxygen partial pressure of the oxygen-rich and nutrient-poor blood is 90 mmHg. The venous blood has an oxygen partial pressure of about 40 mmHg [2]. The liver consists of about $1.25×10^6$ lobules and these from about $2.0×10^5$ hepatocytes [3]. The oxygen consumption of the liver is expressed with up to 6.5 ml/min and 100 g of liver tissue.

With these data, the O_2 consumption of a liver cell calculated from the following formula:

$$q_{o2le} = 6.5\ ml\ /\ (100\ g\ min)\ m_{le}\ /\ V_M = 261\ mmol/h \quad (1)$$

$$q_{o2z} = q_{o2le}\ /\ (N_{ll}\ N_{zll}) = 261\ mmol\ /\ h\ /\ (1.25×10^6\ 2.0×10^5) = 1.04\ pmol\ /\ h \quad (2)$$

This oxygen consumption rate (ocr) of hepatocytes is in the same order of magnitude as published by other groups [4]. The total flow of oxygen is calculated as follows:

$$q_{o2g} = q_{ah}\ c_{o2ah} + q_{pv}\ c_{o2pv} = 677.6\ mmol\ /\ h \quad (3)$$

Here are q_{ah} and q_{pv} the arterial and venous flow rates and c_{o2ah} or c_{o2pv} give the corresponding O_2 concentrations. In the liver lobule is blood flow decreased according to the number of lobules.

$$q_{ll} = q_{bl} / N_{ll} = 1.5\ l / min / 2.0×10^5 = 1.2\ µl / min \quad (4)$$

Means the total flow rate and the O_2 flow, the average O_2 concentration can be determined on the portal field.

$$c_{o2p} = q_{o2g} / q_{bl} = 7.5\ mol / m^3 \quad (5)$$

The concentration of oxygen in central vein calculated by the following equation:

$$c_{o2cv} = (q_{o2g} - q_{o2le}) / q_{bl} = 4.6\ mol / m^3 \quad (6)$$

The size of the liver lobule is average 1.25 mm in diameter and 1.75 mm in height [5]. The shape is polygonal with most 6 vertices. The supply of the liver lobule takes place via the portal fields. The central vein is supplied with 3 till 6 portal fields [6].

In order to supply the liver lobule with cell culture medium, for example, the same amount of O_2 must be provided. Since only approx. 0.18 mol/m^3 oxygen dissolves in the medium under incubator conditions, the flow rate must be increased accordingly [4].

$$q_{me} = q_{o2ll} / \Delta_{o2me} = q_{o2z}\ N_{ll} / \Delta_{o2me} = 0.209\ µmol / h / 0.174\ mol / m^3 = 20\ µl / min \quad (7)$$

1.2. Micro Thermoforming

Micro structuring process such as micro thermoforming was applied to produce the topographical patterns on the laminated heavy ion track etched polycarbonate (PC) membrane with a thickness of approx. 55 µm. The laminate is formed from two polycarbonate films. A thinner non-porous film (6 µm Pokalon, Lofo Hightech film, Weil a.R., Germany) and a thicker porous film (50 µm PC, 1 Mio. pores/cm^2, it4ip, Louvain-la-Neuve, Belgium). Both are laminated in an upstream process to form a semi-finished product. The hot lamination is carried out at approx. 190°C with a speed of 0.8 m/min. A constant contact pressure of approx. 300 N at a roll width of 300 mm must be ensured. Together with a 50 µm FEP film (holscot Europe, Breda, Netherlands), which serves as a force transduction layer, precut pieces from 100 × 140 mm^2 where placed inside the thermoforming mold. The FEP layer was placed underneath the PC laminate, the sealed side of the laminate faced the microstructures inside the mold.

The mold was closed by the thermoforming machine (WLP 1600S, WICKERT Presstech, 76829 Landau i.d.Pf., Germany). During the Movement of the mold a vacuum was applied to the foil stack to keep it in place. After closing, the mold was heated to 100°C, the process chamber was overall evacuated and heated up further to 158°C. reaching this temperature a annealing time of 30 s was allowed to relax the foil. Subsequently the mold was completely closed and cooling immediately started. While closing completely a pressure of 55 bar was applied in order to allow precise molding. Afterwards, the mold was kept under vacuum from the convex side and pressure from the concave side and cooled down. Reaching the release temperature of 80°C the pressure was release, the vacuum was vented and the mold starts to open. Finally, the newly structured PC membrane was carefully released from the mold and the FEP layer was peeled off.

The so micro structured PC laminate was transferred to an etch bath an etched for 10 to 15 minutes in 5 N NaOH- solution add 70°C. Afterward the structures were rinsed carefully and inspected via optical microscopy.

1.2.1. Microcontact printing and chemical functionalization

For the selective patterning of ECM molecules, proteins were applied only to the top features of PDMS stamp by inverted µCP. A glass coverslip was covered with collagen type I (C3867, Sigma) diluted to a concentration of 200 µg/ml, laminin (L2020, Sigma) diluted to working concentration of 100 µg/ml or fibronectin (F1141, Sigma) diluted to 50 µg/ml in distilled water. In the meantime, the PDMS stamp was treated (150 W, 120 s) with oxygen plasma to achieve hydrophilicity and to ensure good transfer of proteins from a glass surface to the stamp features. An oxidized stamp was then turned upside

down such that the features were facing down and gently placed onto the protein-coated glass. To ensure a good contact of the stamp with the glass surface, the stamp was shortly pressed down using tweezers. After 30 min the stamp was carefully removed and finally used as a mold in the microthermoforming process. Therefore, microstructuring and microcontact printing were performed in one step. First, the patterned face of the PDMS stamp was placed in contact with the PC membrane, while a 50 μm FEP foil was placed underneath the porous foil. The asssembled stack was then inserted into the chamber of the microthermoforming machine and heated to 100°C; the process chamber was completely closed and evacuated. At 158°C the foil was tempered for 30 s, and a pressure of 40 bar was applied to stretch the PC membrane over the PDMS mold. Afterward, the machine was cooled down and vented to atmospheric pressure. Finally, the newly formed PC film was carefully released from the PDMS mold and FEP foil to obtain patterned MatriGrid®.

1.3. Analysis of the MEA signals

The evaluation software "DrCell" from the FH Aschaffenburg (BioMEMS Lab, Prof. Thielemann) was used to analyze the 2D and 3D MEA measurements of rat cortex neurons and neurospheres. "DrCell" is a MatLab toolbox. While the *.rhd files created by the NBS measuring system for the 3D-MEA measurements could be loaded and evaluated in "DrCell" without further conversion, the *.mcd files generated by the Multi Channel Systems measuring system must be used via the Multi Channel Data Manager and the MatLab Add-On "McsMatlabDataTools" can be converted to the *.h5 file format.

For the analysis of the measurement signals, a fixed threshold value (threshold) was defined in "DrCell" in order to distinguish the cell signals from the noise. The cell signal must exceed this threshold to be counted as a spike. As the simplest method of calculating this threshold, 5 times the standard deviation of the measurement signal was used as the threshold. Using "DrCell", the 2D and 3D MEA measurements were filtered prior to analysis in order to remove artefacts (e.g. 50 Hz mains hum) from the signals. The signal needs to be filtered because the threshold increases with the level of noise, so that the threshold is higher for measurements with a lot of noise than any spikes that may occur. The comparison of an unfiltered and filtered measurement file is shown in figure 1.

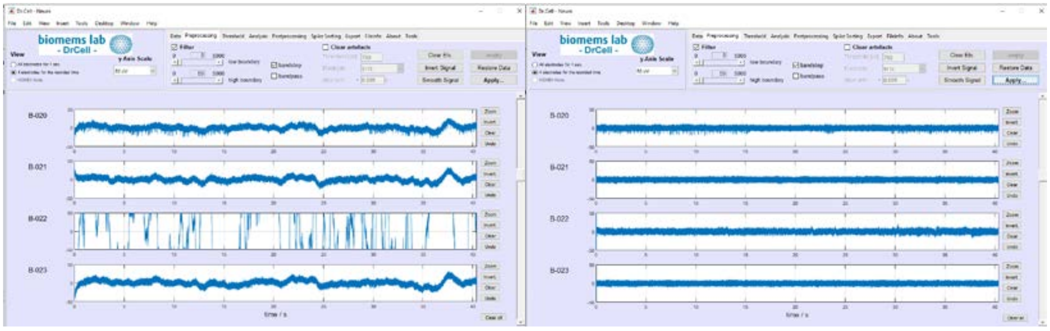

Figure 1: (left) unfiltered signals from different electrodes, (right) filtered signals using "DrCell" software

The signals from the individual MEA electrodes were analyzed using spike and burst events. Since spikes on different electrodes of a measurement can be positive (µV) or negative (-µV), both negative and positive spikes were counted in the analysis. Bursts were calculated using the Baker et al. [7]. The counted events are indicated by green (spikes) and yellow (bursts) triangles in the signal and are shown as numerical values for each electrode (see Figure 2).

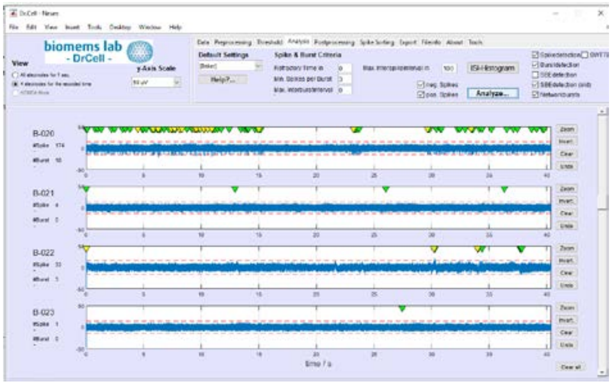

Figure 2: Example of a signal analysis using "DrCell"

To display the spike and burst events, the voltage curves of the individual electrodes are displayed over the measurement time. The signal strengths of the individual electrodes can be compared with one individual electrodes can be related. This makes it possible to identify possible false positive events from the spikes. Such false positive signals can be caused by vibrations in the laboratory (closing the door, movement near the sensor, manipulation of the sensor, etc.) or insufficient contact between the measuring system and the MEA when installing the MEA. If the same spike or burst sequence of non-adjacent electrodes is identical, a fault can be assumed. In addition to determining false positive signals, the raster plot can also be used to derive a dependency between the electrodes (stimulus transmission).

1.4. Automated cultivation and drug administration

The culture unit was constructed with the following properties: utilization of the in house-existing micro bioreactor and MatriGrid® scaffolds (Fig. 7 and Fig 2E in manuscript), active perfusion of the 3D cell culture and automated medium change and sampling of the culture medium for the purpose of analysis with the optional dilution. The fluidic network of the culture unit is divided into two parts (Fig. 10, in manuscript). The first part comprises the circulation loop, the bioreactor and the fresh medium reservoir. It is required to work under aseptic conditions; therefore this part is removable. The cell culture, supported on the MatriGrid®, can be inserted into the bioreactor and the whole circulation loop can be filled with culture medium under the clean bench. The second part

of the fluidic network was designed to handle sample or the waste medium from the bioreactor and the sterile operating conditions are not required. To avoid biomarkers absorption or adsorption on the surface of fluidic pathways, the design avoids using silicon materials like PDMS. The optimal flowrate for the perfusion of 3D grown HepaRG culture on MatriGrid® was found to be 12 µl/min. The culture unit was operated at 37°C in the CO_2-incubator.

The system developed and described in this work represents a robust 3D cell culturing tool with automated medium change and automated biomarker analysis intended for daily laboratory use to support long-term experiments with minimal contamination risk and additional labor-saving benefits. Careful selection of the fluidics construction materials and optimization of fluidic components and operations allowed the construction of a flow-through based ELISA system which can offer automated reliability compared to a traditional manual assay format. We demonstrated the functionality of this system with polycarbonate-scaffold cultured HepaRG organoids, which, due to their hepatofunctional properties, can be used adequately for 2D-cultivated HepaRG cells in toxicity assays. In addition, automated 3D perfusion of HepaRG cultures appears to improve hepatofunctionality compared to 2D cultured HepaRG cells for use in repeated dose toxicity studies with longer experimental durations. Taking all these benefits into account, the system has the potential to be utilized at larger scale levels with significantly higher throughput. This would greatly accelerate the development and testing of new drug therapies while simultaneously reducing cost and helping to improve existing alternatives to *in vivo* animal models for DILI evaluation.

1.5. 3D Hepato MatriGrid®

3D organotypic cell culturing was performed in porous polycarbonate scaffolds named MatriGrid®. Production and quality control of the scaffolds was described in detail in [36]. Porous polycarbonate films are patterned using our micro thermoforming technology, with only the cavity being porous [41]. Scaffold consists of a rectangular 50-micron thick biocompatible polycarbonate (PC) piece with a micro structured seeding area of 5×5 mm^2 and 187 cavities.

1.5.1. upcyte and HepaRG cell culture

For long term culture experiments (up to 28 days) upcyte hepatocytes (donor 422A) were seeded in different cell numbers (50.000, 100.000, 150.000 cells) in MatriGrids® and cultured in hepatocyte performance medium (HPM). For comparison of 2D and MatriGrid® upcyte hepatocyte culture, 100.000 cells (donor 10_03) were seeded and grown for 7 days. Undifferentiated HepaRG cells were grown for maintenance in Williams' Medium E containing 10% fetal bovine serum (FBS), 5 µg/ml insulin, 5×10^{-5} M hydrocortisone hemisuccinate, 2 mM glutamine, 100 U/ml penicillin and 100 µg/ml streptomycin at 37°C in a cell incubator at 95% relative humidity and 5% CO2. HepaRG cells were seeded at a density of 50,000 cells in collagen coated MatriGrid®-scaffolds and grown for 28 days. After seeding, cells were cultured for 2 weeks in the maintenance medium. Thereafter, cells were shifted to differentiation medium on day 14 (supplemented with 1% DMSO). Medium was renewed every 2 days.

1.6.Lung MatriGrid®

1.6.1. Seeding procedure

For the 3D co-culture model of the alveolus, alveolar epithelial cells (A549) and endothelial-like cells (EA.hy926) were cultured separately on both sides of the MatriGrid®. Epithelial cells are located on the apical side of the MatriGrid®, while endothelial cells are located on the basal side of the MatriGrid®.

Prior to cell colonization, the basal side of the MatriGrid® was coated with collagen type I to enable better adhesion of the endothelial cell line. For this purpose, inserts were placed in Petri dishes with the MatriGrids® facing upwards and 25 µl of the collagen solution was added to the basal side of the MatriGrid® and incubated for 1 h under the sterile

bench. After incubation, excess of collagen was discarded the basal side of the MatriGrid® was rinsed several times with DMEM+ to remove collagen residues. Collagenization of the apical side of the MatriGrid® was not found to be necessary for A549 cells.

The cell lines used were cultured to confluence in small cell culture flasks in 5 ml DMEM (A549) or 5 ml DMEM+ (EA.hy926) up to a maximum of the 20th passage with an initial cell count of 1000000 cells per flask.

The colonization of both sides of the MatriGrid® is shown schematically in Figure 13A (in manuscript), where the two cell lines are trypsinized from the culture bottle only shortly before being applied to the MatriGrids® in order to keep the stress on the cells as low as possible. The cell numbers were determined on the Casy CellCounter & Analyser (Innovatis).

The first step was the colonization of the basal side of the MatriGrid® with EA.hy926 cells. For this purpose, the inserts were placed in sterile Petri dishes with the MatriGrid® facing upwards, as for collagenization. According to the cell number, one drop of cell suspension (25 µl) was added to the basal side of the MatriGrid® (Fig. 13A top left, in manuscript). During a 4-hour incubation in closed Petri dishes in the incubator, the cells adhered to the MatriGrid®. Subsequently, the inserts were transferred into 24-well MTPs. The individual wells were filled with 400 µl DMEM+ to ensure nutrient supply of the basal EA.hy926 cells. On the apical medium-free side of the MatriGrid®, the epithelial cells were seeded with 25 µl cell suspension (Fig. 13A top right, in manuscript). After another 2 h incubation for the adhesion of A549 cells on the apical side of the MatriGrids® in the incubator, the 3D co-culture was transferred to a liquid-liquid-interface (LLI) culture for 24 h. For this purpose, another 400 µl were–added to the apical side of the MatriGrids®. By transferring to new MTPs with 400 µl DMEM+, the 3D co-culture model is subsequently created as an ALI culture. In this culture variant, it is cultured with daily medium changes until biological endpoint analyses were performed.

1.7. NEUROGRID®

1.7.1. Brainbits

Rat cortices from Brainbits® (E18 Sprague Dawley Rat, Brainbits®, USA) were used as the source for primary neurons. These specially prepared rat cortices are not frozen for shipment but are shipped in HEB medium. The dissociation of the cortices to obtain the cortical neurons is only performed in the laboratory after shipment. This avoids damage to the neurons from freezing and thawing of the cells.

For the dissociation, the supplied protocol (Appendix 1) was carried out, which is based on the method of Brewer and Price (Brewer1993). In the first step, the cortices were transferred from the HEB medium to the dissociation solution (2 mg/ml papain in Hibernate E medium without calcium (HE-Ca medium)) using a Pastuer pipette. Care was taken to pipette as little HEB medium as possible. This was followed by a 10-minute incubation in a water bath at 30°C, during which the vial was gently inverted every 5 minutes. After the incubation with as little dissociation solution as possible, the cortices were transferred back to the HEB medium with the Pasteur pipette and immediately dispensed there for about 1 min. If the neurons were visibly detached from the cortex, the supernatant was transferred to a 15 ml reaction vessel after the non-disperse pieces had been removed. Approx. 50 µl of the suspension were left in the vial in order to remove the remains of the cortex. The transferred suspension was centrifuged at 1100 rpm and the supernatant discarded. The cell pellet was resuspended in 1 ml NbActic1 medium. The number of cells in the cell suspension was determined using the tryban blue assay and the corresponding number of cells was seeded.

1.7.2. Neurospheres

Neurospheres (NS) from neuronal stem cells (NSC) were used as the second neuronal cell material. The NS were differentiated from human embryonic stem cells (hEMC) into NSCs by the GSI Helmholtz Center for Heavy Ion Research in Darmstadt. All experiments were performed according to the German Stem Cell Act (approval registry numbers

3.04.02/0069 and 3.04.02/0069-E01, issued by the Robert-Koch-Institute, Zulassungsstelle für Anträge nach dem Stammzellgesetz, Berlin, Germany). These NSCs in turn were then cultivated at the GSI, but also in the laboratories of the TU Ilmenau with the following protocol to NS.

In the first step, 6-well MTPs were coated with 1 ml Geltrex® under the sterile bench for 1 hour. Unused coated MTPs were sealed with parafilm and stored in a refrigerator at 4°C. Stored plates must be warmed under the sterile bench for 15-20 min before use. The vials containing the frozen NSCs were thawed in a 37°C water bath. When only small pieces of ice were left, the entire content of a vial was transferred to 8 ml of cold NEM without growth factors (bFGF and EGF) (15 ml reaction vessel) and centrifuged at 200 g for 5 min. Then the supernatant was discarded and the cell pellet was resuspended with 2 ml of warm NEM with growth factors (bFGF and EGF). The cell number was measured using the Casy Cell Counter, for which 50 µl of the cell suspension were pipetted into a 10 ml Casy tube. The NSCs were seeded with a cell density of 2×10^6 cells per well on the coated 6-well MTP and supplied with 2 ml NEM with growth factors. The incubation took place at 37°C in the incubator, the cell culture medium was carried out every 2 days by replacing the entire amount of medium.

The NSCs were passaged once a week when the cell culture area was fully grown. For this purpose, the medium was completely discarded and the wells of the 6-well MTP were washed with 1 ml warm PBS. After the washing step, the cells were detached with 0.5 ml Accutase for 4 min. The detachment of the cells can be checked with the microscope. The detached cells were taken up in 1 ml PBS, after which the cell culture area was rinsed several times with this amount of PBS. The cell suspension was then centrifuged at 1221 rpm for 4 min. The supernatant was discarded and the cell pellet was taken up in 1 ml NEM, well resuspended and the cell count was determined on the Casy Cell Counter by transferring 50 µl of the cell suspension into a Casy tube with 10 ml Casyton. The NSCs were then filled into coated 6-well MTPs at a cell density of 10^6 cells per ml cell culture medium, pipetted with 2 ml NEM and incubated at 37°C.

For the actual creation of the neurospheres, T25 cell culture flasks were coated with Anti-Adherence Rinsing Solution (company) in advance. For this purpose, 1 ml of the Anti-Adherence Rinsing Solution was pipetted into the T25 flask and the entire cell culture surface was wetted by swirling. Coating was then carried out in the incubator for 15 minutes. In contrast to the passage to obtain the NSC culture, the cells after detachment were taken up in NS/B27 medium instead of NEM. The Anti-Adherence Rinsing Solution was completely removed from the cell culture flasks prior to loading the NSCs and replaced with 5 mL of NS/B27 medium with growth factors. The optimal cell density for creating Neurospheres is 1.1×10^6 NPCs per bottle. The incubation also took place at 37°C. It should be noted here that the closure of the cell culture flasks must be slightly open so that the cell culture is supplied with sufficient oxygen. Therefore, the cell culture flasks were incubated with the neck of the flask towards the back of the incubator to avoid contamination. The neurospheres were cultured for at least 3-4 weeks before they were applied to MEAs or MatriGrid®s to avoid falling apart during transfer. The longer the neurospheres are cultivated, the larger they become. The size of the NS should be adapted to the structure or the experiment. The medium was changed once a week. For this purpose, the cell culture flasks were removed from the incubator and placed upright under the sterile bench with the cap closed. The NS were then pipetted into 15 ml reaction vessels together with the entire medium from the bottles and the NS were allowed to settle for 15 min. The NS/B27 medium was then discarded except for a residual amount of 1 ml and replaced with 4 ml of fresh NS/B27 medium. The neurosphere suspension was pipetted back into the respective cell culture flask and incubated with the lid open.

If GSI neurospheres were used to study growth on MatriGrid®s and measurements on 2D and 3D MEAs, the NS were transported to Ilmenau. The transport took place between the 11th and 19th passage, with the number of passages depending on the quality of the neurospheres. The larger the neurospheres, the stronger the cell network and the

more suitable they are for transport. Smaller neurospheres, in turn, have a stronger tendency to stick to surfaces. The transport took place in NS/B27 medium without medium additives. After transport, the NS were transferred to fresh NS/B27 medium with medium additives and were cultivated for a maximum of 7 days without changing the medium before being applied to MEAs and MatriGrid®s.

1.8. TissGrid®

1.8.1. Explant preparation and superfusion/perfusion in TissGrid®-containing bioreactors

Placenta explants were taken from fresh placentas shortly after birth. Uniformity of placenta explants was ensured by weighing. Up to the perfusion experiments placenta explants were stored in warm DMEM medium plus 10% FBS and Pen/Strep solution. One explant was placed in one TissGrid®, which was subsequently inserted in a bioreactor. Explants were flooded for 14 days either with perfusion or superfusion with flow rates of 100 µl/min. Medium was changed automatically every 24 h. After 14 days explants were characterized by live dead staining.

1.9. General methods for characterization of cell health, -function and -morphology

1.9.1. Live/dead staining

Neurospheres after outgrowth of neurons on structured and unstructured PC foil for 11 days and placenta explants after 14 days superfusion or perfusion were incubated with culture medium contain 2 µM Calcein and 4 µM EthDIII for 45 min. Medium was discarded and fresh PBS solution was added. Images were captured with an OLYMPUS laser scanning microscope FV1000 (Olympus, Germany).

1.9.2. Immunofluorescence staining

Upcyte hepatocytes (donor 10_03) were grown on coverslips (2D) or in HepatoGrids® for 7 days. A549 cells were grown in Lung MatriGrids® for 5 days in ALI culture. Neurospheres were precultured for 5 days and then transferred to unstructured or structured PC foil. After 13 days incubation neurospheres were then stained. Cells or neurospheres were washed twice with PBS and fixed for 15 min with 4% paraformaldehyde. After permeabilization with 0.25% Triton in PBS for 5 min, cells were blocked with 5% BSA in PBS for 30 min. Upcyte cells were incubated with ActinStain- phalloidin for 30 min in the dark and afterwards incubated over night with a mouse anti human ZO-1 antibody followed by incubation with secondary Alexa Fluor 647 labeled goat anti-mouse antibody.

A549 cells were incubated with mouse anti human ZO-1 antibody or with rabbit anti human E-Cadherin antibody over night. Subsequently incubation with either AlexaFluor 647-labeled goat anti mouse Antibody or AlexaFluor 488-labeled goat anti rabbit antibody occurred for 1 h at RT in the dark. Neurons outgrown from neurospheres were labeled with mouse anti human anti ßIII tubulin antibody and with rabbit anti human MAP2 antibody over night followed by incubation with AlexaFluor 647-labeled goat anti mouse Antibody or AlexaFluor 488-labeled goat anti rabbit antibody for 1 h at RT in the dark. Cells were mounted in Mowiol containing DAPI. Images were captured with an OLYMPUS laser scanning microscope FV1000 (Olympus, Germany).

1.9.3. LDH Activation Assay

To perform the LDH assay, first buffers, LDH Substrate Mix, NADH Standard, LDH Positive Control and the sample medium were thawed and brought to RT. Then the plate was loaded with standard, sample and positive control. Black 96-well MTPs with clear bottoms were used and samples and standards were measured in duplicate. standards were measured in duplet. For this purpose, a standard series was prepared from six concentrations and, depending on plate occupancy, 50 µl of the concentrations were pipetted onto the 96-well MTP.

In addition, a NADH standard from Cayman Chemical was used, which was supplied in a vial containing 1.5 mg of lyophilisate was supplied. The lyophilizate was dis-

solved in 1808 µl of demineralized water to generate a 1.25 mM standard. For each measurement, a standard series was generated. For this purpose, 25; 24; 23; 22; 21; 20 µl of buffer were first applied in duplicate to a 96-well plate. With the standard, the wells were filled to a volume of 25 µl, resulting in concentrations of 0; 1.25; 2.5; 3.75; 5 and 6.25 mM. For the samples, 20 µl of LDH Assay Buffer was placed in the appropriate wells and 5 µl of medium supernatant collected from perfusion experiments was added to the buffer. In addition, a positive control was also measured, for which 24 µl LDH Assay Buffer was mixed with 1 µl LDH Positive Control. Shortly before the measurement 25 µl Master Reaction Mix (24 µl LDH Assay Buffer and 1 µl LDH Substrate Mix) were pipetted onto each well used. Subsequently, absorbance was measured immediately, after 5 minutes and after 10 minutes at 450 nm.

1.9.4 Albumin-ELISA

A commercially available human albumin ELISA quantitation kit (Bethyl laboratories, Montgomery, TX, USA) was used for albumin measurement. The instructions of the albumin-ELISA quantitation kit were followed. Albumin concentrations of medium supernatants from 48 h cultivation (experiments with HepatoGrids®) or from 24 h perfusion (repeated drug administration) were analyzed by comparison with a concurrently generated calibration curve in the range of 6.25 – 200 ng/ml. Albumin levels were normalized to the total cell number only in the experiments with HepatoGrids®. TMB substrate was used for the MTP ELISA (Immunochemistry Technologies, #6275).

1.10. Nomenclature

q_{bl}	Total blood flow rate (1.5 l/min
q_{ah}	Arterial blood flow rate (500 ml/min)
q_{pv}	Venous blood flow rate (1000 ml/min)
q_{o2le}	O_2 consumption liver
q_{o2z}	O_2 consumption of a hepatocyte
q_{ll}	Total flow per liver lobule
c_{o2ah}	O_2 concentration *arteria hepatica* (8.92 mol/m^3)
c_{o2pv}	O_2 concentration *vena portae* (6.83 mol/m^3)
c_{o2p}	O_2 concentration outside liver lobule (Zone 1)
c_{o2cv}	O_2 concentration inside liver lobule (Zone 3)
N_{ll}	Number of liver lobules
N_{zll}	Number of cells per liver lobule
Δ_{o2me}	Max usable O_2 concentration in culture medium (0.174 mol/m^3)

For the literature citation used in this document, please refer to the main manuscript.

1. Materne, E.-M. Generation of a multi-organ-chip-based liver equivalent for toxicity testing. TU Berlin, 2014.
2. Jungermann, K.; Kietzmann, T. Oxygen: modulator of metabolic zonation and disease of the liver. *Hepatology* **2000**, *31*, 255-260, doi:10.1002/hep.510310201.
3. Kuntz, E.; Kuntz, H.-D. Hepatology textbook and atlas history, morphology, biochemistry, diagnostics clinic, therapy. **2008**.
4. Weise, F.; Fernekorn, U.; Hampl, J.; Klett, M.; Schober, A. Analysis and comparison of oxygen consumption of HepG2 cells in a monolayer and three-dimensional high density cell culture by use of a matrigrid(R). *Biotechnol Bioeng* **2013**, *110*, 2504-2512, doi:10.1002/bit.24912.
5. Lippert, H.; Deller, T. *Lehrbuch Anatomie 204 Tabellen ; [mit dem Plus im Web ; Zugangscode im Buch]*, 8., neu bearb. Aufl. ed.; Elsevier: München, 2011; p. 862 S.
6. Claußen, S.G. Die Sauerstoffversorgung der Leber. Westfälische Wilhelms-Universität Münster, 1994.

7. Baker, R.E.; Corner, M.A.; van Pelt, J. Spontaneous neuronal discharge patterns in developing organotypic mega-co-cultures of neonatal rat cerebral cortex. *Brain Research* **2006**, *1101*, 29-35, doi:https://doi.org/10.1016/j.brainres.2006.05.028.
